# Pulmonary interleukin 1 beta/serum amyloid A3 axis promotes lung metastasis of hepatocellular carcinoma by facilitating the pre-metastatic niche formation

**DOI:** 10.1186/s13046-023-02748-4

**Published:** 2023-07-13

**Authors:** Chong Zhang, Qing Li, Qi Xu, Wei Dong, Chunmei Li, Bin Deng, Jiao Gong, Li-Zhen Zhang, Junfei Jin

**Affiliations:** 1 Guangxi Key Laboratory of Molecular Medicine in Liver Injury and Repair, Affiliated Hospital of Guilin Medical University, Guilin, 541001 Guangxi China; 2 Guangxi Health Commission Key Laboratory of Basic Research in Sphingolipid Metabolism Related Diseases, Affiliated Hospital of Guilin Medical University, Guilin, 541001 Guangxi China; 3 Laboratory of Hepatobiliary and Pancreatic Surgery, Affiliated Hospital of Guilin Medical University, Guilin, 541001 Guangxi China; 4 China-USA Lipids in Health and Disease Research Center, Guilin Medical University, Guilin, 541001 Guangxi China; 5 Department of Urology, The First Affiliated Hospital of Sun Yat-sen University, Guangzhou, 510080 Guangdong China; 6 Department of Laboratory Medicine, Third Affiliated Hospital of Sun Yat-sen University, Guangzhou, 510630 Guangdong China

**Keywords:** IL-1β, SAA3, MMP9, HCC, Pre-metastatic niche

## Abstract

**Background:**

Increasing evidence suggests a vital role of the pre-metastatic niche in the formation of distant metastasis of many cancers. However, how the pre-metastatic niche is formed and promotes pulmonary metastasis of hepatocellular carcinoma (HCC) remains unknown.

**Methods:**

Orthotopic liver tumor models and RNA-Seq were used to identify dysregulated genes in the pre-metastatic lung. *Il1b* knockout (*Il1b*^*−/−*^) mice and lentivirus-mediated gene knockdown/overexpression were utilized to demonstrate the role of interleukin 1 beta (IL-1β)/serum amyloid A3 (SAA3) in the pre-metastatic niche formation and pulmonary metastasis. The potential molecular mechanisms were investigated by RNA-Seq, real-time quantitative PCR (qPCR), western blotting, immunohistochemistry (IHC), flow cytometry, luciferase reporter assay, double immunofluorescent staining and H&E staining.

**Results:**

Accumulation of myeloid cells and upregulation of IL-1β were observed in the pre-metastatic lung of orthotopic liver tumor models. Myeloid cells accumulation and pulmonary metastasis were suppressed in *Il1b*^*−/−*^ mice and Il1r1-silencing mice. Mechanistically, SAA3 and matrix metallopeptidase 9 (MMP9) were identified as potential downstream targets of IL-1β. Overexpression of SAA3 in the lungs of *Il1b*^*−/−*^ mice restored myeloid cells accumulation and pulmonary metastasis of the orthotopic HCC xenografts. Moreover, alveolar macrophages-derived IL-1β dramatically enhanced SAA3 expression in alveolar epithelial cells in an NF-κB dependent manner and increased MMP9 levels in an autocrine manner. Furthermore, SAA3 recruited myeloid cells to the lung without affecting the expression of MMP9 in myeloid cells.

**Conclusions:**

Our study suggests a key role of pulmonary IL-1β and SAA3 in creating a permissive lung pre-metastatic niche by enhancing MMP9 expression and recruiting myeloid cells, respectively, thus promoting pulmonary metastasis of HCC.

**Supplementary Information:**

The online version contains supplementary material available at 10.1186/s13046-023-02748-4.

## Background

Hepatocellular carcinoma (HCC) is a leading course of cancer-related death in the world and its incidence is growing worldwide [1]. Despite advances in cancer treatment, metastasis remains the leading cause of death in cancer patients, highlighting the importance of identifying key molecules in every step of metastasis [2]. Before the arrival of tumor cells, a supportive microenvironment at a distinct organ (termed the pre-metastatic niche) can be induced by the primary tumor [3]. Increasing evidence suggests a vital role of the pre-metastatic niche in the formation of distant metastasis of many cancers, including breast cancer, Lewis lung cancer, melanoma, ovarian cancer, pancreatic cancer, colon cancer and prostate cancer [4, 5]. Pulmonary metastasis is the most common extrahepatic metastasis in patients with advanced HCC, leading to an extremely poor prognosis. However, how the pre-metastatic niche is formed and promotes pulmonary metastasis of HCC remains unknown.

The pre-metastatic niche consists of tumor-mobilized bone marrow-derived cells (BMDCs), the local cells of the host (e.g., fibroblast, macrophages, endothelial cells and epithelial cells), and molecules secreted by them [5–10]. Several characteristics of the pre-metastatic niche are characterized, including recruitment of myeloid cells, increased angiogenesis and vascular permeability, inflammation and immunosuppression [4]. For example, primary tumor-secreted angiopoietin-like 4 (ANGPTL4) and exosomal miR-105 enhance vascular permeability in the pre-metastatic niche [7, 11]. Local endothelial cells-derived serum amyloid A3 (SAA3), which is induced by S100A8 and S100A9, recruits CD11b^+^ myeloid cells and tumor cells into the lung [12]. Activated by primary tumor-derived exosomes, alveolar epithelial cells secrete a number of chemokines that recruit neutrophils to the lung [8]. Interestingly, Gr-1^+^CD11b^+^ cells produce a large amount of matrix metalloproteinase 9 (MMP9) which promotes vascular remodeling and BMDC invasion [6, 13]. In addition, tumor-derived versican creates an inflammatory microenvironment by stimulating the production of TNF-α in myeloid cells [14]. Ovarian tumor-derived inflammatory factors induce neutrophil extracellular traps (NETs) which bound ovarian cancer cells and promote metastasis [15]. Furthermore, IL-10-producing GPX3^+^ alveolar type 2 (AT2) epithelial cells inhibit CD4^+^ T cell proliferation but enhance regulatory T cell generation [9]. Breast cancer-educated alveolar macrophages lessen the number and maturation of lung dendritic cells by modulating TGF-β expression and create an immunosuppressive microenvironment in the lung [16]. Taken together, a complex network between local cells and myeloid cells participates in forming the pre-metastatic niche.

As a proinflammatory cytokine, IL-1β plays important roles in inflammation and cancer development [17]. It activates downstream NF-κB and MAPK signaling pathways by binding to its receptor IL-1R1 which recruits IL-1RAP and subsequent MYD88/IRAK4/TRAF6 [18]. It can induce angiogenesis, promote the production of Th17 cells, and sustain the immunosuppressive function of tumor-infiltrating myeloid-derived suppressor cells (MDSCs) [19, 20]. A clinical study showed that inhibition of IL-1β with its monoclonal antibody canakinumab decreased the incidence of lung cancer in patients with atherosclerosis [21]. However, the role of IL-1β in the formation of pre-metastatic niche is still unclear.

Based on RNA-Seq analysis, we found significant upregulation of Il1b in the pre-metastatic lung of orthotopic mouse HCC models. Moreover, *Il1b* knockout (*Il1b*^−/−^) or I11r1-silencing mice showed impaired formation of pre-metastatic niche and pulmonary metastasis. Mechanism investigation revealed that alveolar macrophages-derived IL-1β stimulated the expression of SAA3 in alveolar epithelial cells in an NF-κB dependent manner and increased MMP9 expression in an autocrine manner. Subsequently, SAA3 attracted MMP9^+^ myeloid cells to form the pre-metastatic niche. Our study suggests IL-1β/SAA3 axis as a critical regulator for the pre-metastatic niche formation of HCC.

## Materials and methods

### Cell lines

Mouse hepatoma cell lines Hepa1-6-luc2 expressing *firefly* luciferase 2 and H22 were gifts from professor Shi-Mei Zhuang’s lab. MLE-12 cell line was a gift from professor Xuefeng Li’s lab. MS1 and RAW264.7 cell lines were purchased from Cellcook (Guangzhou, China). Hepa1-6-luc2, MLE-12 and RAW264.7 were cultured in DMEM (GIBCO, NY, USA) supplemented with 10% fetal bovine serum (FBS), penicillin and streptomycin. H22 was maintained in RPMI 1640 supplemented with 10% FBS, penicillin and streptomycin. MS1 was maintained in DMEM supplemented with 5% FBS, penicillin and streptomycin.

### Mice

*Il1b*^*+/−*^ mice (C57BL/6J background) were bought from GemPharmatech Co., Ltd (Jiangsu, China). Exon4-exon5 of *Il1b* was deleted by CRISPR/cas9 endonuclease-mediated genome editing. C57BL/6J mice were bought from Hunan Slyke Jingda Laboratory Animal Co. LTD (Hunan, China). All mice were kept in specific pathogen-free (SPF) facility with standard food and distilled water.

### Reagents

The primary antibodies used for western blotting included: MMP9 (ab228402, Abcam, USA), β-actin (RM2001, Beijing Ray, Beijing, China).

The primary antibodies used for IHC included: S100A9 (ab242945, Abcam), Ly6G (ab238132, Abcam), CD11b (ab133357, Abcam), F4/80 (70,076 S, CST, Danvers, MA, USA), IL-1β (12,242 S, CST), MMP9 (ab228402, abcam).

The primary antibodies used for immunofluorescence (IF) included: CD11b (ab133357, Abcam), F4/80 (70,076 S, CST), IL-1β (12,242 S, CST), MMP9 (ab228402, abcam), SPC (ab211326, abcam).

The primary antibodies used for flow cytometry included: TruStain FcX anti-mouse CD16/CD32 antibody (Biolegend, 101,319), FITC anti-mouse/human CD11b (Biolgend, 101,205), PE/Cyanine7 anti-mouse Ly-6G (Biolegend, 127,617), Alexa Fluor 647 Rat anti-mouse S100A9 (BD Pharmingen, 565,833) and PE anti-mouse IL-1β (Thermo, 12-7114-80).

Other reagents included: Cultrex Basement Membrane Extract (R&D, Minneapolis, MN, USA); recombinant murine IL-1β (Peprotech, Rocky Hill, USA), murine SAA3 (General Biol, China); IKK 16, JNK-IN-7, Losmapimod and Ravoxertinib (MedChemExpress, Monmouth Junction, NJ, USA).

### Vector construction

To generate pCDH-Saa3, the protein-coding sequence of mouse *Saa3* gene was cloned into the lentiviral vector pCDH-CMV. To generate pCDH-shIl1r1 and pCDH-shNC, a short hairpin RNA targeting mouse *Il1r1* (shIl1r1) or non-target shRNA (shNC) was cloned to the lentiviral vector pCDH-U6. Sequences of shRNAs are listed in Supplementary Table 1.

Transcription start site (TSS) of mouse *Saa3* is designated as +1 (chr7: 46,717,500). Firefly luciferase reporter vector p-(−1.6k/+0.2k) was constructed by cloning the −1600 to +200 bp sequence of mouse *Saa3* gene into the pGL3.0-basic vector (Promega, USA). To generate p-∆NF-κB plasmid, which had a deletion of the predicted NF-κB binding site (−948 to −939 bp upstream of *Saa3*), reverse PCR was utilized based on the p-(−1.6k/+0.2k) plasmid by KOD-Plus-Mutagenesis Kit (TOYOBO, Japan). All vectors were validated by sequencing.

### Tumor metastasis model

Male C57BL/6J mice weighing 18–20 g were utilized for the orthotopic liver xenograft model. Hepa1-6-luc2 (7*10^5^ cells) or H22 (7*10^5^ cells) cells were suspended in a mixture of DMEM and Matrigel (1:1, R&D Systems) and implanted into the left hepatic lobe of mice. Mice were harvested at indicated timepoints. The tumor volume (V) was calculated by the formula V = (Length * Width^2^) /2. To evaluate the metastasis burden of Hepa1-6-luc2, the whole lung tissues were harvested and subjected to qPCR analysis for the mRNA level of luc2, which represented the relative number of Hepa1-6-luc2. In addition, metastasis burden of H22 and Hepa1-6-luc2 xenografts was also evaluated by calculating the number of metastatic foci under microscope after H&E staining. For H&E staining, thirty sections throughout the whole lung tissues were used for each mouse. All animal experiments were conducted in accordance with the Guide for the Care and Use of Laboratory Animals (NIH publications no. 80 − 23, revised 1996) and approved by the institutional ethical guidelines of Guilin medical college.

### Flow cytometry

The mice were perfused with PBS to remove blood cells in the lung tissues before sacrificing. Then the lung tissues were dissociated into single-cell suspension by using the Lung dissociation kit (Miltenyi Biotec, Germany) according to the manufacturer’s guidelines. Red blood cells were removed using 1× RBC Lysis buffer (eBioscience, 00-4333-57). Cells were blocked using TruStain FcX anti-mouse CD16/CD32 antibody. For the flow cytometry analysis of CD11b/Ly6G/S100A9, the cells were stained with FITC anti-mouse/human CD11b, PE/Cyanine7 anti-mouse Ly-6G, followed by fixation, permeabilization and staining with Alexa Fluor 647 Rat anti-mouse S100A9. For the flow cytometry analysis of IL-1β, the cells were stained with PE anti-mouse IL-1β after fixation and permeabilization. FACS analysis was performed on Agilent NovoCyte Quanteon Flow Cytometer.

### Lentivirus production and lentiviral infection in the lungs of mice

HEK293T cells were used to produce lentiviruses. For each dish, 4 µg of pCDH, pCDH-Saa3, shNC or shIl1r1 plasmid and packaging plasmids (3 µg psPAX and 1 µg pMD2.G) were co-transfected into HEK293T cells using Lipofectamine 3000. After 48 and 72 h, viruses were harvested and concentrated using lentivirus concentration kit (Genomeditech, Shanghai, China). For in vivo lentiviral infection, lentiviruses (5*10^7^ vg in 60 µL PBS) were given to the lungs of mice by intratracheal injection after anesthetization.

### RNA-Seq

To identify the dysregulated genes in the pre-metastatic lung niche, total RNAs from lung tissues of control mice and mice bearing Hepa1-6-luc2 cells orthotopically for 2 weeks were isolated. RNAs from 3 individual control mice and tumor-bearing mice were applied to RNA-Seq by Beijing Tsingke Biotech Co., Ltd. (Beijing, China). “DESeq2” R package was used to analyze the differentially expressed genes (DEGs) between two groups. The criterion of false discovery rate (FDR) < 0.01 and |log_2_ fold-change (FC)| >= 1 was used [22].

To identify genes that were regulated by IL-1β in tumor-bearing lungs, total RNAs from lung tissues of WT mice and *Il1*b^−/−^ mice bearing Hepa1-6-luc2 cells orthotopically for 2 weeks were isolated. RNAs from 3 individual WT and *Il1b*^−/−^ tumor-bearing mice were mixed equally, respectively, and then applied to RNA-Seq by Tsingke Biotechnology Co.Ltd. (Tianjin, China). “EdgeR” R package was employed to conduct analysis and DEGs were screened with the cutoff of |log_2_ (FC)| >= 1 and FDR < 0.01 [23].

### Enrichment analysis of KEGG pathway and construction of PPI network

Kyoto Encyclopedia of Genes and Genomes (KEGG) enrichment analysis of the DEGs was performed with “clusterProfiler” R package [24]. FDR < 0.05 was used in the study. To further select the crucial modules and genes, protein-protein interaction (PPI) analysis of the DEGs was performed by the STRING database (https://cn.string-db.org/) with a medium confidence score more than 0.4. Then a PPI network was created using Cytoscape software and the key modules were constructed by Molecular Complex Detection (MCODE) module based on the cutoff score >= 2 [25].

### Analysis of mRNA expression by qPCR

Total RNAs were isolated from cell lines or lung tissues using Trizol reagent and then converted to cDNA by using the Hifair® II 1st Strand cDNA Synthesis Kit (gDNA digester plus) (Yeasen, Shanghai, China). Realtime qPCR was performed by using the 2× SYBR Green qPCR Master Mix (Low ROX) (Bimake, Shanghai, China) on the LightCycler® 96 Instrument according to the manufacturer’s guidelines. The relative levels of target genes were normalized to that of GAPDH (an internal control) to calculate the 2^−ΔΔCt^ value. The primers used in the study are listed in Supplementary Table 2.

### Transfection of siRNA duplexes

Cells were transfected with siRNA duplexes using Lipofectamine 3000 according to the manufacturer’s guidelines. All siRNA duplexes were purchased from Beijing Tsingke Biotech Co., Ltd., and the sequences of siRNA duplexes are listed in Supplementary Table 2.

### Immunohistochemical (IHC) and double immunofluorescence (IF) staining

As described previously, formalin-fixed paraffin-embedded tissues were used. Antigen retrieval was performed by microwave heating in EDTA buffer (pH 9.0) for Ly6G, S100A9 and MMP9 or in 10 mM citrate buffer (pH 6.0) for CD11b, IL-1β and F4/80. Sections were incubated at 4 °C overnight with rabbit polyclonal antibody against CD11b, Ly6G, S100A9 and MMP9 or with mouse polyclonal antibody against IL-1β, then immunostained by SignalStain® DAB Substrate Kit (CST). Then five random fields of each section under the Leica microscope at 200× were photographed, the number of CD11b, Ly6G, S100A9 and IL-1β positive cells were presented by the average number of positive cells per field.

For double IF staining of IL-1β and F4/80 or CD11b, anti-mouse IgG (Alexa Fluor® 555 Conjugate) and anti-rabbit IgG (Alexa Fluor® 488 Conjugate) were used.

For double IF staining of MMP9 and SPC or CD11b, the Double-Fluorescence immunohistochemical mouse/rabbit kit (Immunoway, Suzhou, China) was used.

### Luciferase reporter assay

Luciferase activity was measured using the dual-luciferase reporter assay system (Promega). Plasmid pRL-TK (Promega) which expressed *Renilla* luciferase was utilized as an internal control to correct the differences in transfection. For transfection, 10 ng pRL-TK and 50 ng pGL3.0-basic/p-(−1.6k/+0.2k)/p-∆NF-κB were co-transfected into MLE-12 cells in a 48-well plate using Lipofectamine 3000 (Thermo). After 24 h, cells were treated with or without IL-1β (10 ng/ml). Cells were harvested 24 h later and luciferase activity was measured using Tecan Spark multimode reader (Switzerland).

### Isolation of bone marrow-derived cells (BMDCs)

Briefly, bone marrow femurs from mice were isolated and placed into a sterile cell culture dish. Both femur ends were cut with a sharp scissor, the marrow was flushed out into a 15 mL conical tube with complete RPMI 1640 medium. All marrow was passed through a 40 μm cell strainer and collected in a 50 mL tube. All cells were spined down at 500 g for 4 min at 4 °C. The red blood cells were lysed with 3 mL red blood cell lysis buffer for 1 min on ice, then 27 mL 1× PBS was added to stop the reaction. The BMDCs were then collected and maintained in complete RPMI 1640 medium.

### Gelatin zymography

RPMI 1640 without FBS was used to harvest the supernatant of BMDC. Proteins from the supernatant were separated by a 7.5% acrylamide gel containing gelatin. The gel was then washed with washing buffer, incubated with incubation buffer, stained with staining solution, and incubated with destaining solution until bands could be seen clearly.

### Detection of chemotactic activity of MLE-conditioned medium by transwell assay

MLE-12 cells (1*10^5^) were seeded in a 24-well plate and stimulated with or without IL-1β (10 ng/mL) for 24 h, then freshly isolated BMDCs (2*10^5^) were added into the 5-µm transwell and then put into the well containing MLE-12 cells. After 12 h, the migrated cells were fixed by methanol and stained with crystal violet. Total number of migrated cells from five random fields (100×) is presented for each sample.

### Statistical analysis

Data from at least three independent experiments are shown as the mean ± SEM. Unless otherwise mentioned, the differences between two groups were analyzed by unpaired or paired Student’s t test. *P* < 0.05 was considered as the criterion of statistical significance in all experiments. *, *P* < 0.05; **, *P* < 0.01; ***, *P* < 0.001; ****, *P* < 0.0001; ns, not significant.

## Results

### The pre-metastatic niche is formed during pulmonary metastasis of HCC

**Fig. 1:**
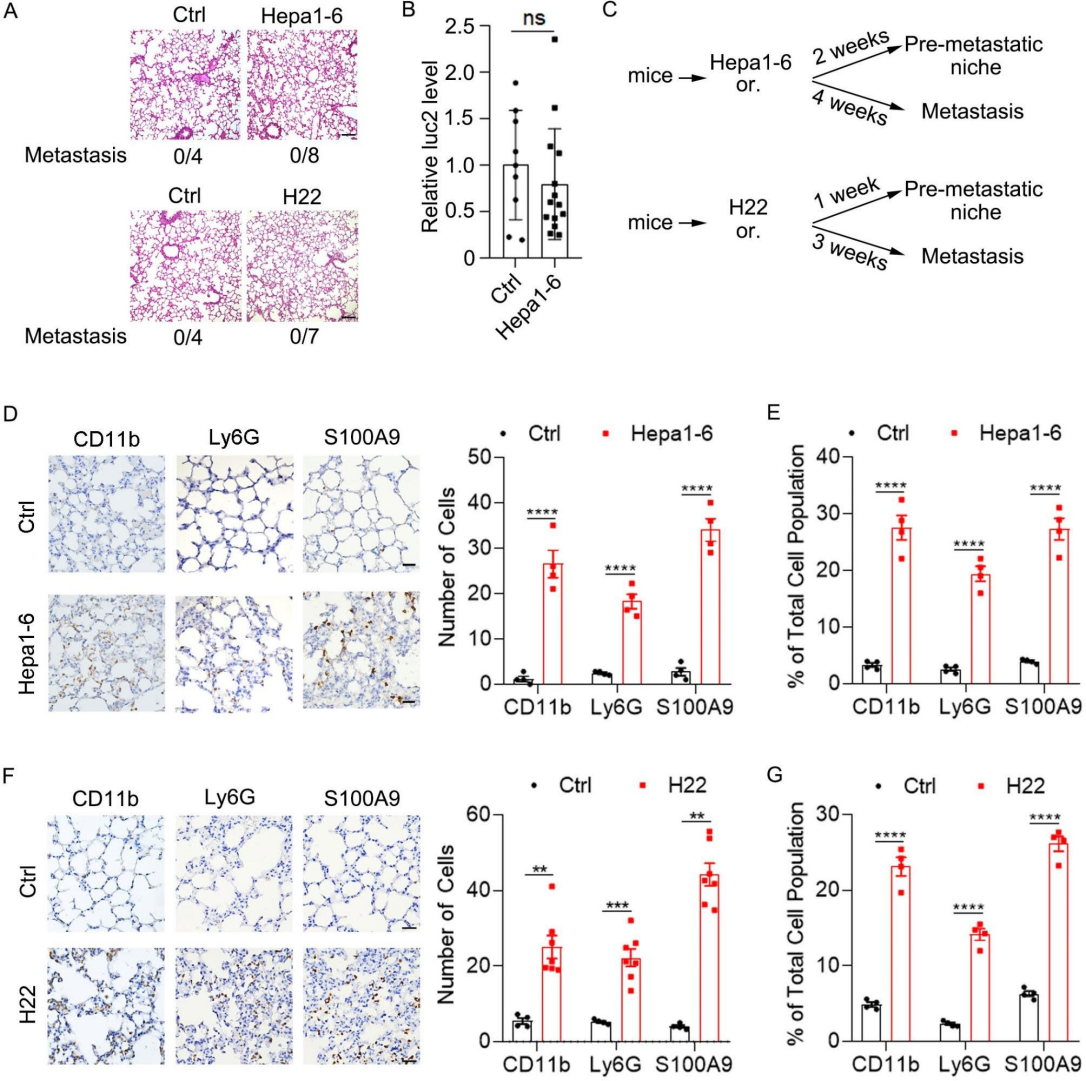
Orthotopic HCC development induces a pre-metastatic niche in the lung. (**A**-**B**) Pulmonary metastasis was not observed 2 weeks after orthotopic Hepa1-6- implantation or 1 week after orthotopic H22 implantation. (**A**) Detection of pulmonary metastasis 2 weeks after orthotopic implantation of matrigel or Hepa1-6 cells (upper) and 1 week after orthotopic implantation of matrigel or H22 cells (lower) in the liver by H&E staining. Scale bar, 100 μ**m**. (**B**) Detection of luc2 by qPCR in the lung of mice 2 weeks after orthotopic implantation of matrigel or Hepa1-6 cells in the liver. (**C**) Schematic for the procedures of Hepa1-6 and H22 xenograft mouse models. Or., orthotopic implantation. (**D**-**E**) Increased numbers of myeloid cells and inflammatory cells in the pre-metastatic lung of orthotopic HCC xenografts. IHC staining (**D**) and flow cytometry analysis (**E**) of CD11b^+^, Ly6G^+^ and S100A9^+^ cells in the lung of mice 2 weeks after orthotopic implantation of Hepa1-6 cells in the liver. Scale bar, 25 μm. (**F**-**G**) IHC staining (**F**) and flow cytometry analysis (**G**) of CD11b^+^, Ly6G^+^ and S100A9^+^ cells in the lung of mice 1 week after orthotopic implantation of H22 cells. Data are displayed as the mean ± SEM; unpaired Student’s t test (**B**, **D**, **E**). **, *P* < 0.01; ***, *P* < 0.001; ****, *P* < 0.0001; ns, not significant

To determine the pre-metastatic phase of HCC xenografts, we detected whether HCC cells entered the lung at early timepoints after orthotopic implantation of HCC cells. Based on our experience, H22 cells grew faster than Hepa1-6-luc2 (Hepa1-6 cell line expressing *firefly* luciferase 2, thereafter named Hepa1-6) in vivo. Therefore, we detected the existence of HCC cells in the lung 2 weeks after Hepa1-6 implantation or 1 week after H22 implantation. As expected, H&E staining showed that no tumor cells were detected in the lung of mice bearing Hepa1-6 for 2 weeks or bearing H22 for 1 week (Fig. 1A). Consistently, qPCR analysis of luc2 which labeled Hepa1-6 cells showed a similar result (Fig. 1B). Therefore, the pre-metastatic niche and metastasis of Hepa1-6 and H22 in the study were detected at indicated timepoints as shown (Fig. 1C). Accumulation of myeloid cells and inflammation has been reported in the pre-metastatic niche of many cancers. Consistently, we found increased numbers of CD11b^+^ (myeloid cells), Ly6G^+^ (neutrophils) and S100A9^+^ (mainly secreted by inflammatory cells) cells in the pre-metastatic lung of mice bearing Hepa1-6 cells and H22 cells as shown by immunohistochemical staining (IHC) and flow cytometry (Fig. 1D and G and Supplementary Fig. 1). Collectively, these results imply that a pre-metastatic niche is formed during pulmonary metastasis of HCC and may participate in the colonization of HCC cells to the lung.

### IL-1β is indispensable for the pre-metastatic niche formation and pulmonary metastasis of HCC

**Fig. 2:**
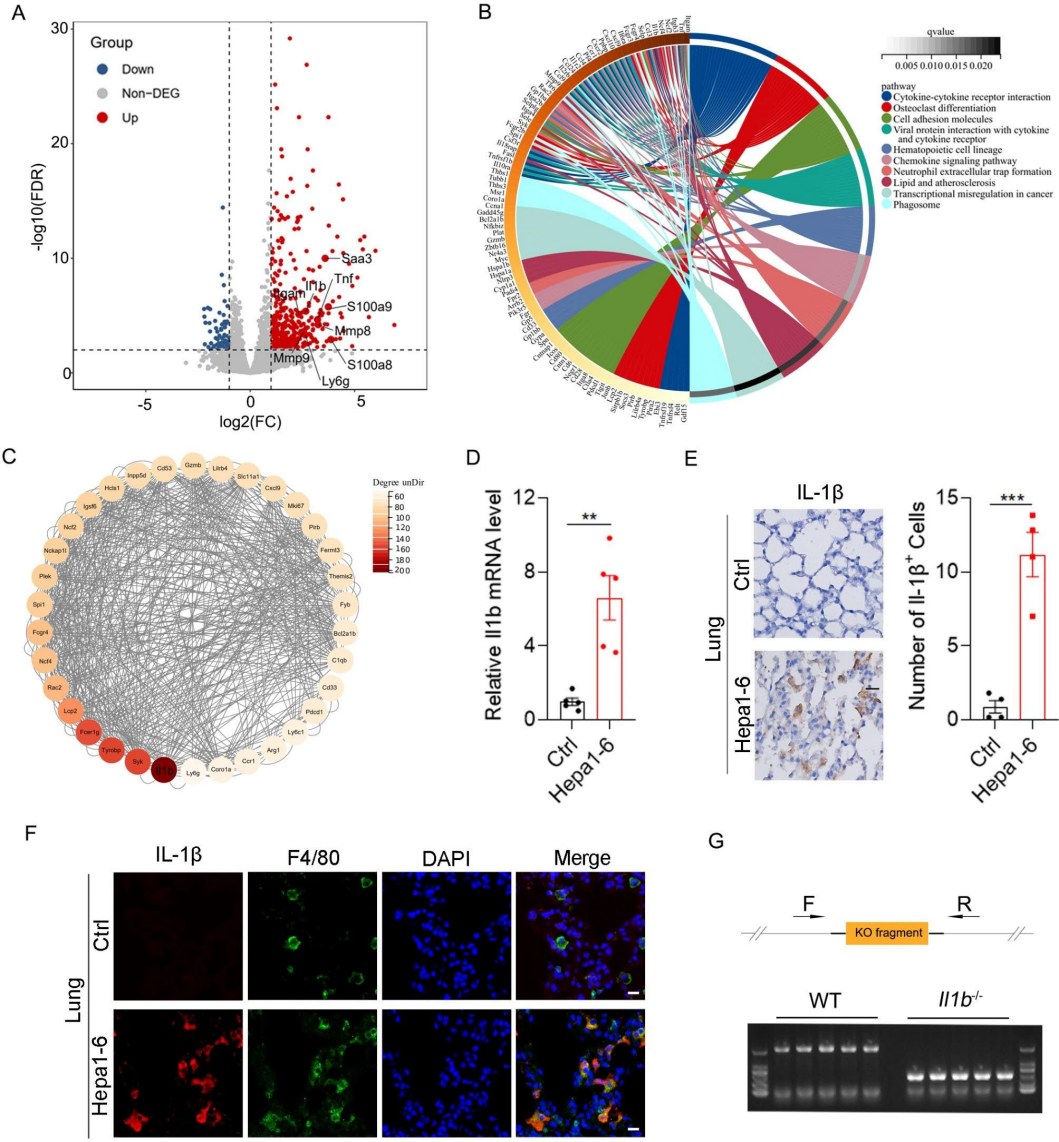
Increased Il1b in the pre-metastatic lung of orthotopic HCC xenografts. (**A**) Volcano plot showing dysregulated genes (Hepa1-6 vs. Ctrl) in the lungs of mice 2 weeks after orthotopic implantation of Hepa1-6-luc2 cells or matrigel in the liver. (**B**) KEGG analysis of dysregulated genes in the pre-metastatic lung of Hepa1-6-luc2 orthotopic xenograft. (**C**) The network of the top one important module was visualized by cytoscape. (**D**-**E**) Increased expression of Il1b in the pre-metastatic lung of HCC. Detection of Il1b in the lungs of mice 2 weeks after orthotopic implantation of matrigel or Hepa1-6-luc2 cells in the liver by qPCR (**D**) and IHC (**E**). Scale bar, 25 μm. (**F**) Co-localization of IL-1β and F4/80 in the pre-metastatic lung. Double immunofluorescence staining of IL-1β (red) and F4/80 (green) in the pre-metastatic lung. Scale bar, 10 μm. (**G**) Genotyping of *Il1b*^*−/−*^ mice. Upper, schematic diagram of primer design for genotyping; lower, agarose gel electrophoresis of the PCR products from WT and *Il1b*^*−/−*^ mice. Data are displayed as the mean ± SEM; unpaired Student’s t test (**D**, **E**). **, *P* < 0.01; ***, *P* < 0.001

To identify the genes that are required for the pre-metastatic niche formation, we performed RNA-Seq to screen the genes that were dysregulated in the lungs of mice bearing Hepa1-6 cells for 2 weeks (Supplementary Material  1). A total of 428 differentially expressed genes (DEGs), including 360 upregulated and 68 downregulated genes, were screened out with the criterion of |log2 FC| >= 1 and FDR < 0.01 (Fig. 2A and Supplementary Fig. 2). Consistent with increased numbers of CD11b^+^, Ly6G^+^ and S100A9^+^ cells in the pre-metastatic niche, the mRNA levels of Itgam (CD11b), Ly6g, S100a9 were also elevated (Fig. 2A). Similar with previous studies, the mRNA levels of Saa3, Mmp9, S100a8 and Tnf were significantly upregulated in the pre-metastatic niche (Fig. 2A). The KEGG analysis of DEGs identified “cytokine-cytokine receptor interaction” as the most enriched pathway in the pre-metastatic niche (Fig. 2B). To further reveal the hub genes involved in the formation of pre-metastatic niche, DEGs were subjected to protein-protein interaction (PPI) analysis, and the PPI network was presented by Cytoscape software. Then the module with the highest score was identified using MCODE, in which Il1b was screened as the most important hub gene based on the interaction with all DEGs as indicated by the biggest degree value (Fig. 2C). As a vital proinflammatory cytokine, Il1b mRNA was notably increased in the pre-metastatic niche (Fig. 2A). Therefore, we focused on the role of Il1b in the pre-metastatic niche formation of HCC.

To further confirm the increased expression of Il1b, qPCR was performed and similar results were obtained (Fig. 2D and Supplementary Fig. 3A). Consistently, IHC staining and flow cytometry showed that IL-1β^+^ cells were increased in the pre-metastatic niche (Fig. 2E and Supplementary Fig. 3B). Next, we explored the cellular origin of IL-1β, double immunofluorescence staining showed that IL-1β co-stained with F4/80 (a macrophage marker) but not CD11b (Fig. 2F and Supplementary Fig. 3C), implying that IL-1β was produced by alveolar macrophages in the pre-metastatic niche.

**Fig. 3:**
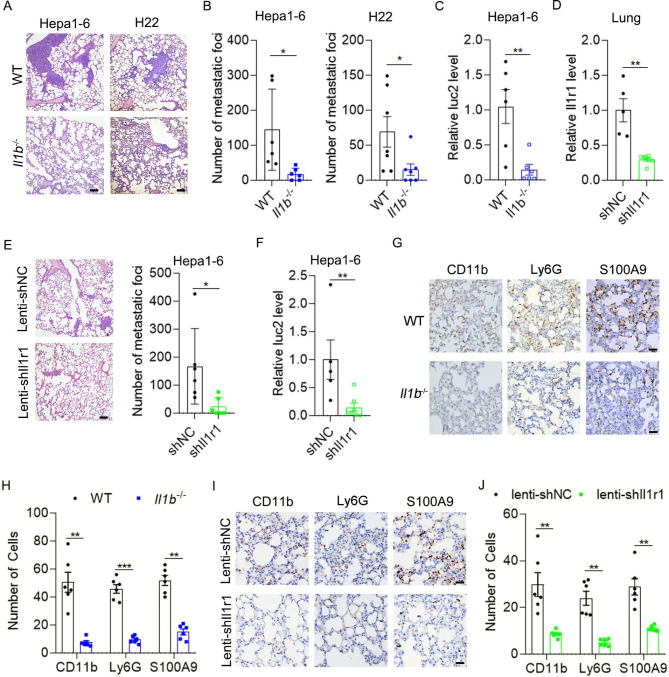
IL-1β/IL-1R1 signaling promotes the pre-metastatic niche formation and pulmonary metastasis of HCC xenografts. (**A**-**C**) Pulmonary metastases of HCC xenografts were reduced in *Il1b*^*−/−*^ mice. Hepa1-6 cells (A and **B**, left panel, and **C**) or H22 cells (**A** and **B**, right panel) were implanted orthotopically in the liver of WT and *Il1b*^*−/−*^ mice, and pulmonary metastases were detected 4 weeks or 3 weeks later by H&E staining, respectively. Scale bar, 100 μm. For (**C**), pulmonary metastasis was detected by qPCR analysis of luc2. (**D**) Intratracheal administration of lenti-shIl1r1 viruses decreased Il1r1 mRNA level in the lung. (**E**-**F**) Silencing of Il1r1 in the lung decreased pulmonary metastases of HCC xenografts. For (**D**-**F**), lenti-shCtrl or lenti-shIl1r1 viruses were given intratracheally to the lung of WT mice. After 3 days, mice were orthotopically injected with Hepa1-6 cells. Il1r1 mRNA level and pulmonary metastases were detected 4 weeks later. Pulmonary metastasis was detected by H&E staining (**E**) and qPCR analysis of luc2 (**F**), respectively. (**G**-**J**) Accumulation of myeloid cells and inflammatory cells in the pre-metastatic lung was suppressed in *Il1b*^*−/−*^ mice (**G**-**H**) and mice infected with lenti-shIl1r1 viruses in the lung (**I**-**J**). For (**G**-**J**), two weeks after orthotopic implantation of Hepa1-6 cells, mice were sacrificed and IHC staining of CD11b^+^, Ly6G^+^ and S100A9^+^ cells in the lung were performed. Scale bar, 25 μm. Data are displayed as the mean ± SEM; unpaired Student’s t test (**B**-**F**, H and **J**). *, *P* < 0.05; **, *P* < 0.01; ***, *P* < 0.001

Next, we explored whether IL-1β was involved in the pulmonary metastasis of HCC. The *Il1b*^*−*/−^ mice were generated and genotyping of *Il1b*^*−/−*^ mice was shown in Fig. 2G. Orthotopic xenografts of HCC cells were performed in wild-type (WT) and *Il1b*^*−/−*^ mice. Interestingly, primary tumor volume was similar between WT and *Il1b*^*−/−*^ mice (Supplementary Fig. 4A and B), but pulmonary metastasis was significantly decreased in *Il1b*^*−/−*^ mice compared with WT mice as shown by H&E staining and qPCR analysis of luc2 (Fig. 3A-C). IL-1β binds to its receptor IL-1R1 and activates the downstream signaling pathways. Then, we silenced the expression of Il1r1 in the lung of mice by intratracheal administration of lenti-shIl1r1 lentivirus (Fig. 3D). Silencing of Il1r1 in the lung significantly decreased pulmonary metastases of Hepa1-6 xenografts without affecting primary tumor growth (Fig. 3E and F and Supplementary Fig. 4C). To determine whether IL-1β increased pulmonary metastases by promoting the pre-metastatic niche formation, we detected the numbers of CD11b^+^, Ly6G^+^ and S100A9^+^ cells in the pre-metastatic phase of HCC metastasis. As expected, the numbers of CD11b^+^, Ly6G^+^ and S100A9^+^ cells in the pre-metastatic lung of HCC in *Il1b*^*−/−*^ mice were significantly decreased compared with those in WT mice (Fig. 3G and H and Supplementary Fig. 5). Consistently, silencing of Il1r1 significantly decreased the numbers of CD11b^+^, Ly6G^+^ and S100A9^+^ cells in the pre-metastatic niche (Fig. 3I and J). These results suggest that IL-1β/IL-1R1 signaling plays an important role in the pre-metastatic niche formation and pulmonary metastasis of HCC.

### Saa3 overexpression restores the pre-metastatic niche formation and pulmonary metastasis of HCC in ***Il1b***^***−/−***^ mice

**Fig. 4:**
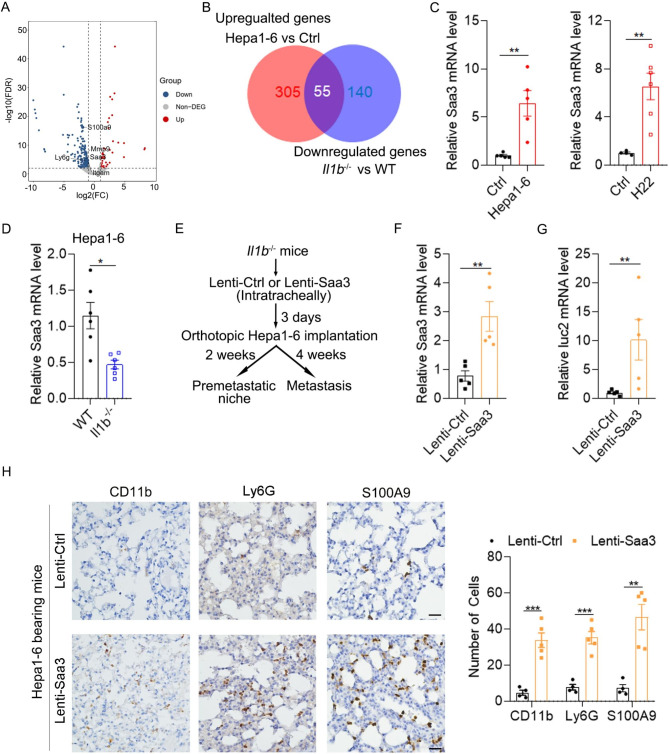
Saa3 is a downstream effector of IL-1β that promotes the pre-metastatic niche formation and metastasis of HCC xenografts. (**A**) Volcano plot showing differentially expressed genes in the pre-metastatic lung of *Il1b*^*−/−*^ mice vs. WT mice. (**B**) Venn diagram showing common and exclusive genes between upregulated genes in the lung of mice implanted with Hepa1-6 vs. matrigel (Ctrl) and downregulated genes in the pre-metastatic lung of *Il1b*^*−/−*^ mice vs. WT mice. (**C**) Increased Saa3 expression in the pre-metastatic lung of HCC xenografts. (**D**) Decreased Saa3 expression in the pre-metastatic lung of *Il1b*^*−/−*^ mice. For C and **D**, qPCR analysis of Saa3 mRNA level was performed 2 weeks after orthotopic implantation of Hepa1-6 cells or 1 week after orthotopic implantation of H22. (**E**) Schematic for experimental design in **F**-**H**. Lenti-Ctrl or lenti-Saa3 viruses were given intratracheally to the lung of *Il1b*^*−/−*^ mice followed by orthotopic implantation of Hepa1-6 cells after 3 days. Two weeks after orthotopic implantation of Hepa1-6 cells, mice were sacrificed and IHC staining was performed. Four weeks later, tumor volume and pulmonary metastasis were detected. (**F**) Intratracheal administration of lenti-Saa3 viruses increased Saa3 mRNA level in the lung of *Il1b*^*−/−*^ mice. (**G**) Overexpression of Saa3 in the lung of *Il1b*^*−/−*^ mice increased pulmonary metastases of HCC xenografts. (**H**) Overexpression of Saa3 in the lung of *Il1b*^*−/−*^ mice increased the numbers of myeloid cells and inflammatory cells in the pre-metastatic lung. IHC staining of CD11b^+^, Ly6G^+^ and S100A9^+^ cells in the pre-metastatic lung were performed. Scale bar, 25 μm. Data are displayed as the mean ± SEM; unpaired Student’s t test (A-F). *, *P* < 0.05; **, *P* < 0.01; ***, *P* < 0.001

To explore the underlying mechanism of IL-1β in promoting the pre-metastatic niche formation, we performed RNA-Seq to identify DEGs expressed in the pre-metastatic lung of WT and *Il1b*^*−/−*^ mice. As shown, we identified 33 upregulated and 195 downregulated DEGs in the pre-metastatic lung of *Il1b*^*−/−*^ mice compared with that of WT mice (Fig. 4A and Supplementary Fig. 6 and Supplementary Material 2). KEGG analysis revealed the DEGs were mainly enriched in “Cytokine-cytokine receptor interaction” (Supplementary Fig. 7). Logically, IL-1β-induced genes were those that were upregulated in the pre-metastatic niche of WT mice (Hepa1-6 vs. Ctrl) but decreased in the pre-metastatic niche of *Il1b*^−/−^ mice (*Il1b*^*−/−*^ vs. WT) (Fig. 4B). Luckily, 55 genes were sorted out and then subjected to PPI analysis (Fig. 4B and Supplementary Fig. 8). Among them, Saa3 and Mmp9 have been reported to play critical roles in the pre-metastatic niche formation. Therefore, we explored whether Saa3 and Mmp9 were involved in the pre-metastatic niche formation induced by IL-1β.

To verify the results of RNA-Seq, we performed qPCR analysis to detect the mRNA level of Saa3. Consistently, Saa3 was increased in the pre-metastatic niche of WT mice bearing HCC xenografts but was decreased in that of *Il1b*^−/−^ mice (Fig. 4C and D). Next, we explored whether restoration of Saa3 in *Il1b*^−/−^ mice increased the pulmonary metastases of HCC (Fig. 4E). As shown in Fig. 4F, overexpression of Saa3 by lentivirus intratracheally significantly increased Saa3 mRNA level in the lung. Moreover, overexpression of Saa3 significantly enhanced pulmonary metastasis of HCC while didn’t affect primary tumor growth in *Il1b*^−/−^ mice (Fig. 4G and Supplementary Fig. 9). SAA3 has been reported to recruit myeloid cells to the lung during the pre-metastatic phase [12]. Consistently, Saa3 overexpression increased the numbers of CD11b^+^, Ly6G^+^ and S100A9^+^ cells in the pre-metastatic niche of *Il1b*^−/−^ mice bearing HCC xenografts (Fig. 4H). These results suggest that SAA3 may be a downstream effector of IL-1β which regulates the pre-metastatic niche formation of HCC.

**Fig. 5:**
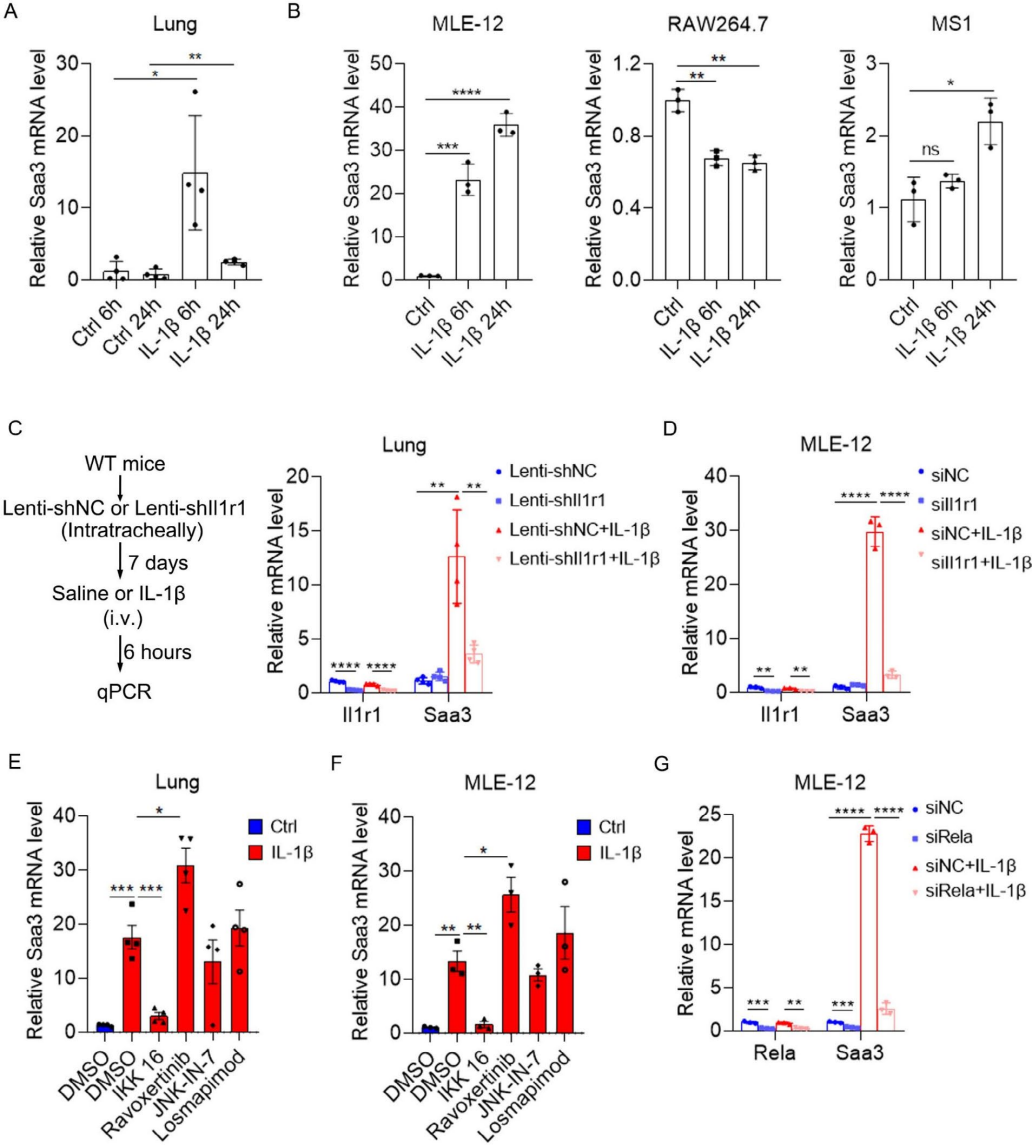
IL-1β induces Saa3 expression in alveolar epithelial cells in an NF-κB dependent manner. (**A**) Intravenous administration of IL-1β induced Saa3 expression in the lung. Detection of Saa3 mRNA by qPCR 6 and 24 h after IL-1β administration (20 ng in 100 ul saline). (**B**) The effect of IL-1β on the expression of Saa3 in different cell lines. Detection of Saa3 mRNA by qPCR 6 and 24 h after IL-1β (10 ng/ml) stimulation on MLE-12, RAW264.7 and MS1 cells. (**C**) Silencing of Il1r1 blocked IL-1β-induced Saa3 expression in vivo. Lenti-shNC or lenti-shIl1r1 viruses were given intratracheally to the lung of WT mice. After 7 days, mice were intravenously given saline or IL-1β (20 ng in 100 ul saline). After 6 h, Saa3 and Il1r1 expression was detected by qPCR. (**D**) Silencing of Il1r1 blocked IL-1β-induced Saa3 expression in MLE-12 cells. Forty-eight hours after siRNA transfection, MLE-12 cells were stimulated with IL-1β (10 ng/ml) for 6 h. Saa3 mRNA level was detected by qPCR. (**E**) The effect of NF-κB, ERK, JNK and p38 inhibitors on IL-1β-induced Saa3 expression in vivo. One hour after intravenous administration of indicated inhibitors, IL-1β (20 ng in 100 ul saline) were i.v. administrated into the mice. Six hours later, lung Saa3 expression was detected by qPCR. (**F**) The effect of NF-κB, ERK, JNK and p38 inhibitors on IL-1β-induced Saa3 expression in MLE-12 cells. MLE-12 cells were pre-treated with indicated inhibitors for 1 h, then stimulated with IL-1β (10 ng/ml) for 6 h. Saa3 expression was detected by qPCR. (**G**) Silencing of p65 blocked IL-1β-induced Saa3 expression in MLE-12 cells. Forty-eight hours after siRNA transfection, MLE-12 cells were stimulated with IL-1β (10 ng/ml) for 6 h. Saa3 mRNA level was detected by qPCR. Data are displayed as the mean ± SEM; (**A**, **E** and **F**) one-way ANOVA with Turkey’s correction. (**B**) one-way ANOVA with Dunnett’s correction. (**C**, **D** and **G**) two-way ANOVA with Turkey’s correction. *, *P* < 0.05; **, *P* < 0.01; ***, *P* < 0.001; ****, *P* < 0.0001; ns, not significant

Then, we explored how IL-1β upregulated Saa3 expression. To test whether IL-1β directly induced the expression of Saa3, we stimulated the lung with IL-1β that was administered by intravenous injection and detected Saa3 mRNA by qPCR. Notably, Saa3 expression was dramatically induced as early as 6 h after IL-1β stimulation (Fig. 5A). Next, we tried to identify the cell type in which Saa3 was induced by IL-1β. Considering that alveolar epithelial cells, endothelial cells, and macrophages were the three most common cell types in the lung, we stimulated a mouse alveolar epithelial cell line MLE-12, a mouse endothelial cell line MS1 and a mouse macrophage cell line RAW264.7 with IL-1β. Only MLE-12 displayed a robust upregulation of Saa3 6 h after IL-1β stimulation while RAW264.7 showed a decreased Saa3 expression and MS1 showed a slightly increased Saa3 mRNA 24 h after IL-1β stimulation (Fig. 5B), suggesting that alveolar epithelial cell may be the major source of Saa3 in IL-1β-stimulated lung. To verify that IL-1R1 mediated IL-1β-induced Saa3 expression, we silenced Il1r1 expression in the lung by lentiviruses and then stimulated the lung with IL-1β. Indeed, silencing of Il1r1 restrained IL-1β-induced Saa3 expression in the lung (Fig. 5C). Consistently, silencing of Il1r1 in MLE-12 cell also blocked IL-1β-induced Saa3 expression (Fig. 5D). IL-1β induces the transcription of downstream genes by activating NF-κB, ERK, JNK and p38 signal pathways, so we repressed these pathways with their inhibitors to identify which signaling pathway mediated IL-1β-induced Saa3 expression. IKK 16, Ravoxertinib, JNK-IN-7, and Losmapimod were used to suppress NF-κB, ERK, JNK and p38 signaling, respectively. Interestingly, only the IKK inhibitor restrained IL-1β-induced Saa3 expression both in the lung and in MLE-12 cell line (Fig. 5E and F). IKK phosphorylates p65 (Rela), which is a component of NF-κB complex, and increases the transcriptional activity of NF-κB. Consistently, silencing of Rela blocked basal and IL-1β-induced Saa3 expression in MLE-12 cell line (Fig. 5G). To confirm that NF-κB binds to the promoter of Saa3, we performed luciferase reporter assays and found that 1600 bp region upstream of Saa3 TSS showed obvious promoter activity, which was increased by IL-1β stimulation (Supplementary Fig. 10). A potential NF-κB binding site was forecasted to be located at this region by using Alibaba and Jaspar algorithm and deletion of this site diminished IL-1β-induced promoter activity (Supplementary Fig. 10). These results suggested that IL-1β induced Saa3 expression in an NF-κB dependent manner.

### MMP9 is involved in the pro-metastatic effect of IL-1β

**Fig. 6:**
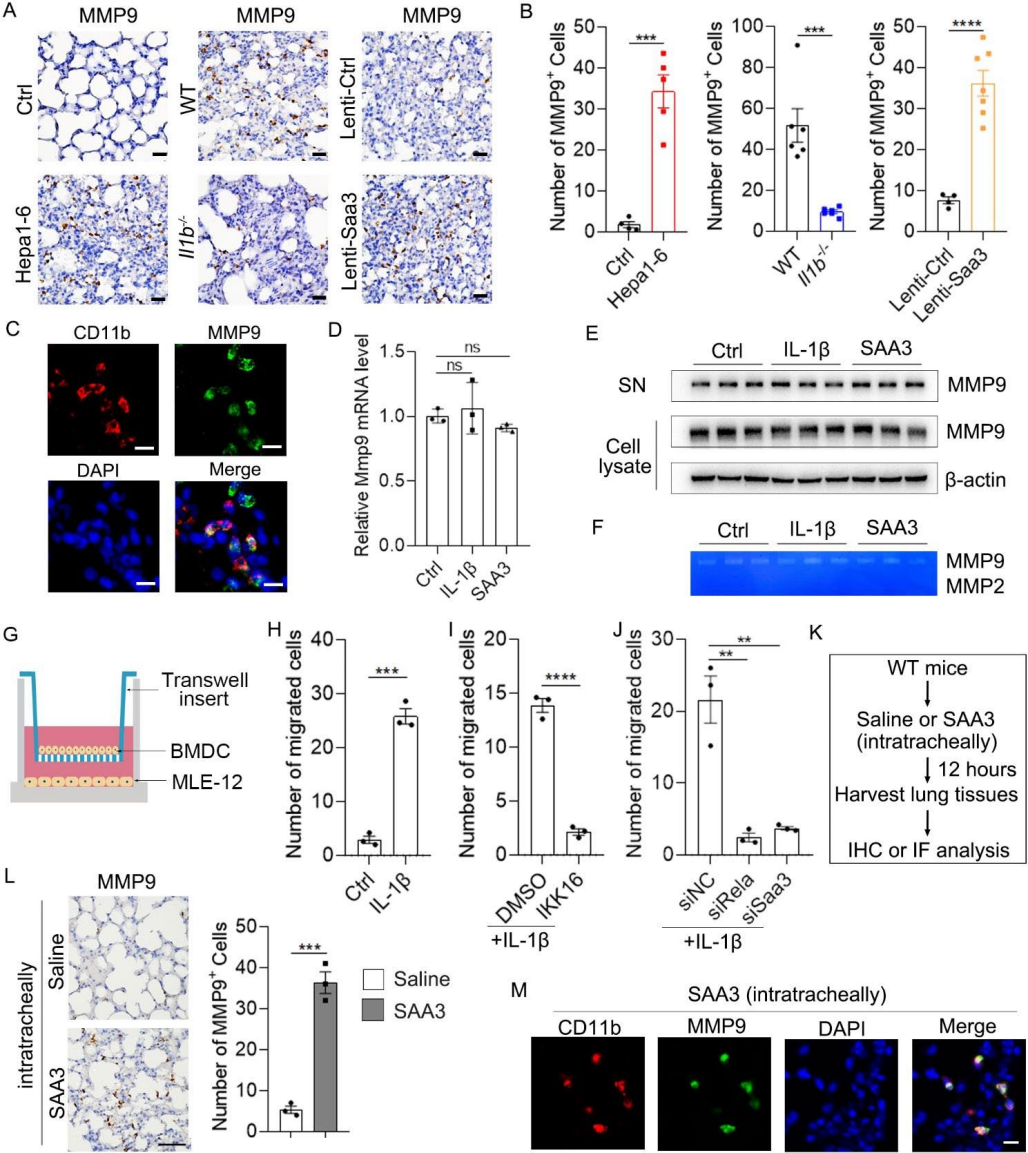
The effect of IL-1β and SAA3 on the recruitment of MMP9^+^myeloid cells and the expression of MMP9 in BMDCs. (**A**-**B**) The number of MMP9^+^ cells in the pre-metastatic lung of Ctrl and Hepa1-6-bearing mice, WT and *Il1b*^*−/−*^ mice, and lenti-Ctrl and Lenti-Saa3 viruses-infected *Il1b*^*−/−*^ mice. Scale bar, 25 μm. (**C**) Colocalization of CD11b and MMP9 in the pre-metastatic lung. Scale bar, 10 μm. (**D**) IL-1β and SAA3 stimulation had no effect on Mmp9 mRNA in BMDCs. Mouse BMDCs were stimulated with IL-1β (10 ng/ml) and SAA3 (3 µg/ml) for 12 h followed by qPCR analysis of Mmp9 mRNA. (**E**-**F**) IL-1β and SAA3 stimulation had no effect on the protein level, secretion and activity of MMP9 in BMDCs. Mouse BMDCs were stimulated with IL-1β and SAA3 for 24 h followed by immunoblotting analysis of MMP9 in cell lysate and supernatant (SN) of BMDCs (**E**). MMP2 and MMP9 activities in the supernatant were detected by gelatin zymography (**F**). (**G**) Schematic for experimental design in **H**-**I**. (**H**) IL-1β enhanced the chemotactic activity of MLE-12-conditioned medium (MCM) for BMDCs. (**I**) IKK 16 blocked the chemotactic activity of IL-1β-stimulated-MCM. (**J**) Silencing of Rela or SAA3 in MLE-12 restrained the chemotactic activity of IL-1β-stimulated-MCM. For **H**-**J**, MLE-12 cells were stimulated with IL-1β for 24 h, and then BMDCs were allowed to migrate for 12 h. (**K**) Schematic for experimental design in **L**-**M**. Saline or SAA3 (1.2 µg in 60 ul saline) were injected intratracheally to the lung of mice. Twelve hours later, lungs were harvested. (**L**) Intratracheal administration of SAA3 recruited MMP9^+^ cells to the lung. MMP9^+^ cells were detected by IHC (left). The number of MMP9^+^ cells was shown (right). Scale bar, 50 μ**m**. (**M**) Intratracheal administration of SAA3 recruited MMP9^+^ myeloid cells to the lung. Double IF staining of CD11b (red) and MMP9 (green) in the lung of mice stimulated with SAA3 intratracheally for 12 **h**. Scale bar, 10 μm. Data are displayed as the mean ± SEM; (**B**, **H**, **I**, **L**) unpaired Student’s t test; (D and J) one-way ANOVA with Dunnett’s correction. **, *P* < 0.01; ***, *P* < 0.001; ****, *P* < 0.0001; ns, not significant

Upregulation of MMP9 has been found in the pre-metastatic niche and promotes pulmonary metastasis of melanoma (B16) and Lewis lung carcinoma (LLC) xenograft. Consistently, increased number of MMP9^+^ cells was also found in the pre-metastatic niche of HCC xenografts (Fig. 6A and B, left, and Supplementary Fig. 11). However, a reduction of MMP9^+^ cells was observed in the pre-metastatic niche of HCC xenograft in *Il1b*^−/−^ mice (Fig. 6A and B, middle). Moreover, overexpression of Saa3 increased the number of MMP9^+^ cells in the pre-metastatic niche of *Il1b*^−/−^ mice (Fig. 6A and B, right). The pattern of MMP9^+^ cells resembled that of CD11b^+^, Ly6G^+^ and S100A9^+^ cells, suggesting that myeloid cells may be the source of MMP9. To confirm this, we co-stained MMP9 with CD11b by immunofluorescence staining. Indeed, a large proportion of MMP9 colocalized with CD11b (Fig. 6C).

MMP9 is a collagenase and functions outside the cell, so we first explored whether IL-1β and SAA3 affected the expression and secretion of MMP9 in BMDCs. Interestingly, neither IL-1β nor SAA3 affected the mRNA and protein level of MMP9 in BMDCs (Fig. 6D and E). Moreover, neither IL-1β nor SAA3 affected the secretion and activity of MMP9 as indicated by the unchanged MMP9 protein level and enzymatic activity in the supernatant of BMDCs (Fig. 6E and F and Supplementary Fig. 12). Interestingly, high MMP9 activity was found in the supernatant of BMDCs while MMP2 activity was undetectable as shown by gelatin zymography (Fig. 6F). These results suggest that IL-1β and SAA3 may be required for the recruitment of MMP9^+^ BMDCs without affecting the expression of MMP9 in BMDCs. Therefore, we further explored whether IL-1β could enhance the chemotactic activity of MLE-12-conditioned medium (MCM) for BMDCs (Fig. 6G). Indeed, IL-1β-stimulated-MCM attracted more BMDCs than control MCM did (Fig. 6H and Supplementary Fig. 13A). Moreover, inhibition of NF-κB by IKK 16 or siRela in MLE-12 blocked the chemotactic activity of IL-1β-stimulated-MCM (Fig. 6I and J and Supplementary Fig. 13B-13C). Consistently, silencing of SAA3 in MLE-12 also restrained the chemotactic activity of IL-1β-stimulated-MCM (Fig. 6J and Supplementary Fig. 13C). Moreover, the chemotactic activity of SAA3 was also verified in vivo. Indeed, intratracheal injection of SAA3, which led to a higher concentration of SAA3 specifically in the lung, recruited MMP9^+^ cells to the lung (Fig. 6K and L). Moreover, MMP9 exactly co-stained with CD11b (Fig. 6M), suggesting SAA3 recruited MMP9^+^ myeloid cells to the lung. These results suggest that IL-1β-induced SAA3 expression may be responsible for the recruitment of BMDCs that expressed a large amount of MMP9 in the pre-metastatic lung.

**Fig. 7:**
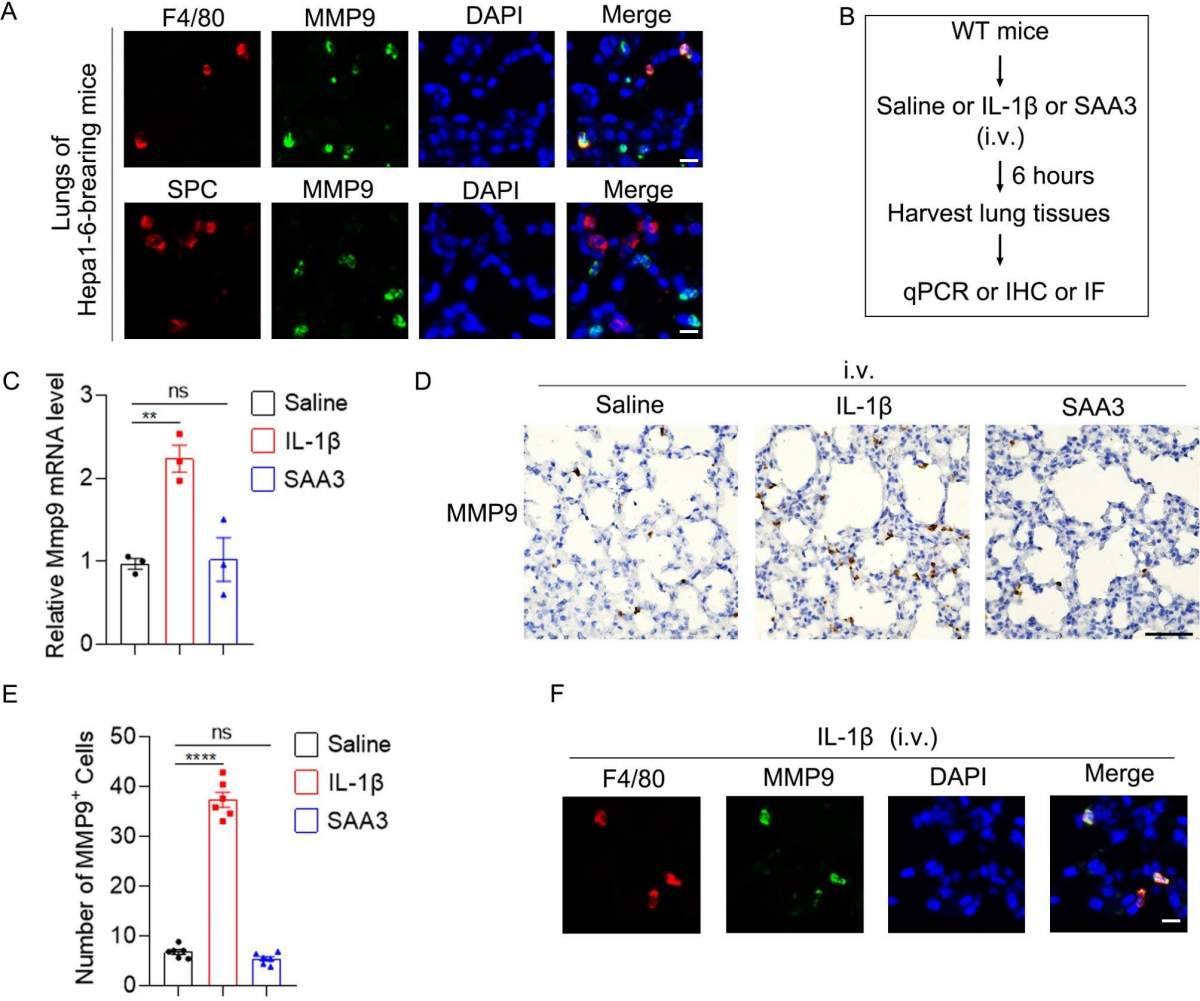
IL-1β increases MMP9 expression in alveolar macrophagesin vivo. (**A**) MMP9 was expressed by alveolar macrophages but not AT2 cells in the pre-metastatic lung. Double IF staining of F4/80 (red) or SPC (red) and MMP9 (green) in the pre-metastatic lung of Hepa1-6-bearing mice. Scale bar, 10 μm. (**B**) Schematic for experimental design in **C**-**F**. IL-1β (20 ng in 100 ul saline) or SAA3 (1.2 µg in 100 ul saline) were injected intravenously. Six hours later, the lungs were harvested. (**C**) Intravenous administration of IL-1β but not SAA3 increased MMP9 mRNA level in the lung. (**D**-**E**) Intravenous administration of IL-1β but not SAA3 increased MMP9^+^ cells in the lung. MMP9^+^ cells were detected by IHC (**D**). The number of MMP9^+^ cells was shown in (**E**). Scale bar, 50 μm. (**F**) IL-1β stimulated MMP9 expression in alveolar macrophages. Double IF staining of F4/80 (red) and MMP9 (green) in the lung of mice stimulated with IL-1β i.v. for 6 h. Scale bar, 10 μm. Data are displayed as the mean ± SEM; (C and **E**) one-way ANOVA with Dunnett’s correction. **, *P* < 0.01; ****, *P* < 0.0001; ns, not significant

Since a portion of MMP9 signal was not co-stained with CD11b (Fig. 6C), we speculated that MMP9 expression in local cells of the lung might be enhanced in mice bearing HCC xenograft. Then, we co-stained MMP9 with F4/80 or surfactant protein C (SPC, maker for AT2 cell). Herein, we found that MMP9 colocalized with F4/80 but not SPC in the lungs of Hepa1-6-bearing mice (Fig. 7A), suggesting that alveolar macrophages also expressed MMP9. Next, we explored whether IL-1β and SAA3 induced MMP9 expression in alveolar macrophages directly in vivo. Intravenous administration of IL-1β but not SAA3 increased Mmp9 mRNA and the number of MMP9^+^ cells in the lung (Fig. 7B and E). Moreover, MMP9 exactly co-stained with F4/80 (Fig. 7F), suggesting that IL-1β stimulated expression of MMP9 in alveolar macrophages. Taken together, these results imply that IL-1β stimulates the expression of MMP9 in alveolar macrophages while IL-1β-induced SAA3 recruits MMP9^+^ BMDCs to the lung.

Collectively, we demonstrate a role of IL-1β/SAA3 axis in the formation of lung pre-metastatic niche and HCC metastasis and suggest this axis as a potential target for anti-metastasis treatment.

## Discussion

**Fig. 8:**
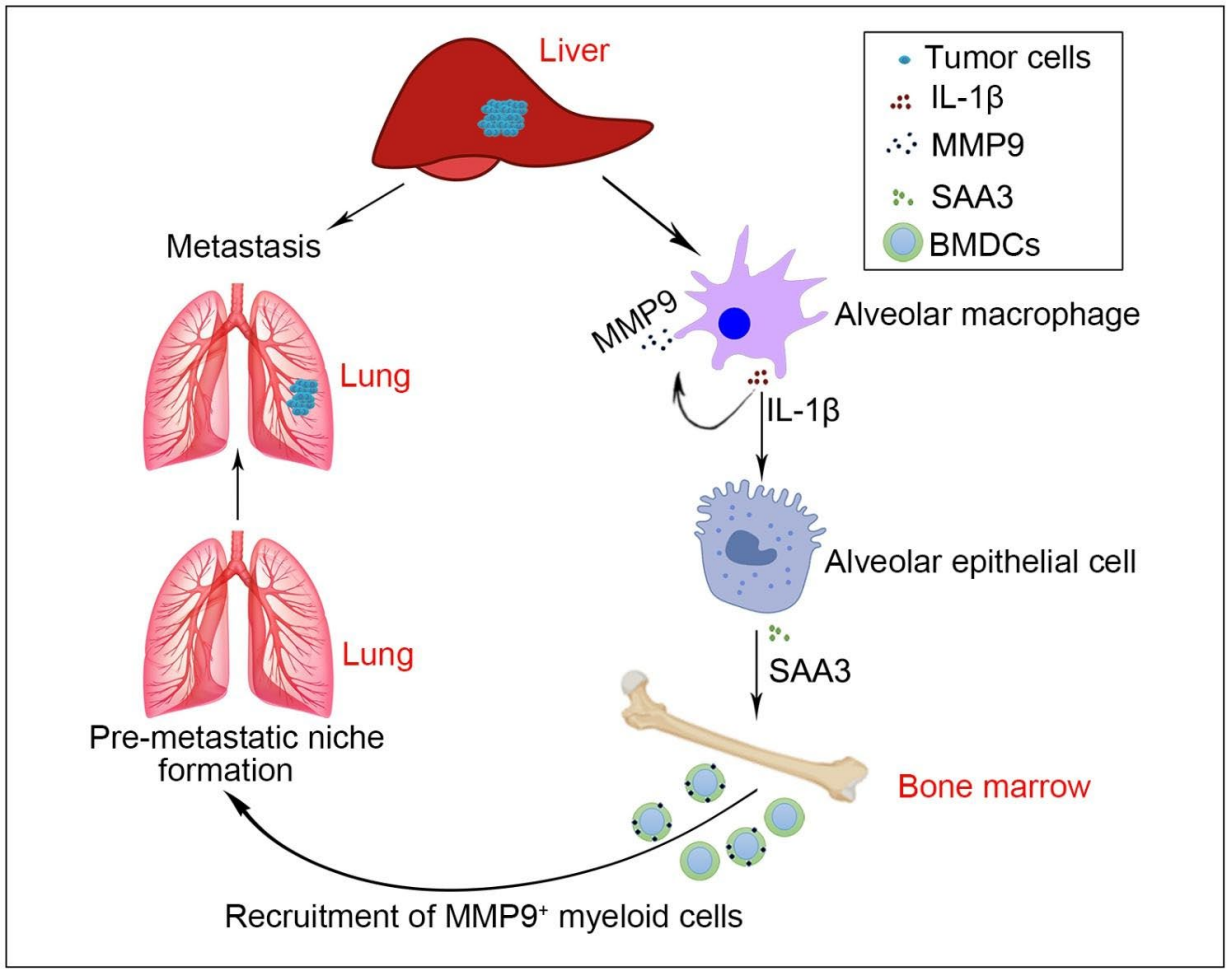
Graphic summary of the role of IL-1β/SAA3 axis in the pre-metastatic niche formation of HCC metastasis. Briefly, hepatoma cells-derived factors upregulate IL-1β expression in alveolar macrophages. On the one hand, IL-1β stimulates MMP9 expression in alveolar macrophages in an autocrine manner. On the other hand, IL-1β enhances SAA3 expression in alveolar epithelial cells. Subsequently, SAA3 recruits MMP9^+^ myeloid cells to the lung. Then, the permissive pre-metastatic niche is formed and promotes pulmonary metastasis of HCC

In this article, we found that Il1b was significantly increased in the pre-metastatic lung of HCC and promoted the pre-metastatic niche formation by upregulating SAA3 level and increasing MMP9^+^ cells, thus facilitating pulmonary metastasis of HCC (Fig. 8). Our findings suggest that targeting IL-1β/SAA3 axis may serve as a potential therapy for preventing against lung metastasis of advanced HCC.

Recruitment of myeloid cells and inflammation are important characteristics of the pre-metastatic niche of many cancers. Consistently, we found increased numbers of CD11b^+^ cells, Ly6G^+^ cells and S100A9^+^ cells in the pre-metastatic lung of HCC. As important pro-inflammatory cytokines, Il1b and Tnf were increased in the pre-metastatic lung of HCC. In addition, increased expression of several immune co-inhibitory receptors, including Ctla4, Tigit and Pdcd1 (PD-1), was observed in the pre-metastatic lung of HCC xenograft (Supplementary Material 1), suggesting the existence of an immunosuppressive microenvironment. Previous study showed that neutrophil extracellular trap (NET) formation in the pre-metastatic omental niche facilitated ovarian cancer metastasis [15]. In our study, KEGG analysis also showed that NET formation was enriched in the pre-metastatic lung (Fig. 2B). Therefore, NET formation in the pre-metastatic lung may be a possible mechanism by which neutrophils promote HCC metastasis.

As a vital pro-inflammatory cytokine, IL-1β promotes cancer development via many different manners in the primary tumor [17, 26–28]. Moreover, blockage of IL-1β improved the efficacy of adoptive NK cell immunotherapy in mitigating lung metastasis [29]. However, we found that Il1b knockout didn’t affect primary tumor growth in HCC xenografts, suggesting that Il1b may promote the malignant transformation of hepatocyte into HCC without affecting the outgrowth of advanced HCC. Herein, we demonstrate an important role of IL-1β in the formation of pre-metastatic lung niche and subsequent metastasis of HCC via a different manner. On the one hand, IL-1β, which was produced by alveolar macrophages, was increased in the pre-metastatic lung. On the other hand, both *Il1b*^*−/−*^ mice and silencing of IL-1R1 in the lung displayed impaired recruitment of myeloid cells to the lung and subsequently decreased pulmonary metastasis of HCC xenografts. Previous studies showed that tumor cell-derived hyaluronan and soluble CD44 could induce the expression of IL-1β in monocyte and macrophages in primary tumor, respectively [30, 31]. Therefore, we speculate that primary HCC-derived factors induce IL-1β expression in alveolar macrophages, which facilitates the recruitment of myeloid cells to the lung.

As an acute-phase apolipoprotein, SAA3 is expressed by several types of cells, including cancer-associated fibroblast (CAF), endothelial cells, macrophages, colonic and alveolar epithelial cells [12, 32, 33]. Microbiota and endotoxin induced SAA3 expression, but the underlying mechanism seems to be independent of NF-κB. One the one hand, LPS induced SAA3 and TNF-α expression in CMT-93 epithelial cells while LPS only induced TNF-α expression in RAW264.7 cells [34]. One the other hand, TNF-α only induced a slight increase of SAA3 in CMT-93 cells and didn’t induce SAA3 expression in RAW264.7 cells [34]. Moreover, inhibition of NF-κB blocked the SAA1 expression while had no effect on SAA3 expression upon S100A4 stimulation [35]. However, we found that IL-1β induced SAA3 expression in alveolar epithelial cells, but not in endothelial cells and macrophages, in an NF-κB dependent manner. This may be explained by different expression of kinase and transcription factors or cofactors between these cells.

SAA3 promotes primary tumor growth and metastasis in different types of cancer [12, 33, 35]. Mechanistically, SAA3 is able to induce the migration of tumor cells and myeloid cells or leukocytes in vitro and in vivo [12, 35]. In addition, SAA3 can induce the expression of MMP3, MMP13 and several cytokines, including G-CSF, S100A8, S100A9 [35]. More importantly, SAA3 enhances survival and suppressive activity of monocytic MDSCs [36]. Herein, we found that SAA3 recruited myeloid cells to the pre-metastatic lung of mice bearing HCC xenograft in vivo. Surprisingly, SAA3 had no effect on the expression of MMP9 in BMDCs in vitro and lung tissues in vivo. However, whether SAA3 has other roles in the pre-metastatic niche formation needs further investigation.

Matrix metalloproteinases (MMPs) are zinc dependent proteolytic metalloenzyme. MMP-9 belongs to the gelatinase family and has the ability to degrade extracellular matrix [37]. Dysregulation of MMP9 contributes to progression of many diseases, including cardiovascular diseases, neurodegenerative diseases and cancer, by promoting vascular remodeling and cell invasion [37–39]. Increased MMP9 is found in macrophages and endothelial cells in the pre-metastatic niche [40]. More importantly, MMP9 produced by myeloid cells promotes vascular remodeling in the pre-metastatic lung. Deletion of MMP9 normalizes aberrant vasculature and diminishes lung metastasis [13]. In addition, less tumor cells after i.v. injection invade the lung and survive in *MMP9*^*−/*−^ mice [40]. Herein, we found MMP9^+^ cells were also increased in the pre-metastatic niche of HCC. On the one hand, alveolar epithelial cell-derived SAA3 recruited MMP9^+^ myeloid cells to the lung. On the other hand, alveolar macrophages-derived IL-1β increased MMP9 expression in an autocrine manner.

## Conclusions

In summary, we discovered that the communication between alveolar macrophages, epithelial cells and myeloid cells orchestrated the formation of the pre-metastatic lung niche and facilitated lung metastasis of HCC. Our study highlights that targeting IL-1β/SAA3 axis may be a potential therapy for anti-metastasis treatment.

## Electronic supplementary material

Below is the link to the electronic supplementary material.

Supplementary Material 1

Supplementary Material 2

Supplementary Material 3

## Data Availability

All data generated or analysed during this study are included in this published article and its supplementary information files.

## List of abbreviations

BMDC: Bone marrow derived cells
H&E staining: Hematoxylin-eosin staining
HCC: Hepatocellular carcinoma
IL-1β: Interleukin 1 beta
JNK: C-Jun N-terminal kinase
MDSC: Myeloid-derived suppressor cells
MMP9: Matrix metallopeptidase 9
NETs: Neutrophil extracellular traps
qPCR: Real-time quantitative PCR
Saa3: Serum amyloid A3
TGF-β: Transforming growth factor beta
TNF-α: Tumor necrosis factor alpha
TSS: Transcription start site
