# Supplementary material for: Pulmonary interleukin 1 beta/serum amyloid A3 axis promotes lung metastasis of hepatocellular carcinoma by facilitating the pre-metastatic niche formation
Source: J Exp Clin Cancer Res. 2023 Jul 13;42:166. doi: 10.1186/s13046-023-02748-4 (PMC10339487; doi:10.1186/s13046-023-02748-4)
Supplement: Supplementary file 3 — Supplementary Material 3 [file 13046_2023_2748_MOESM3_ESM.docx]

**Supplementary Figures and Figure legends**


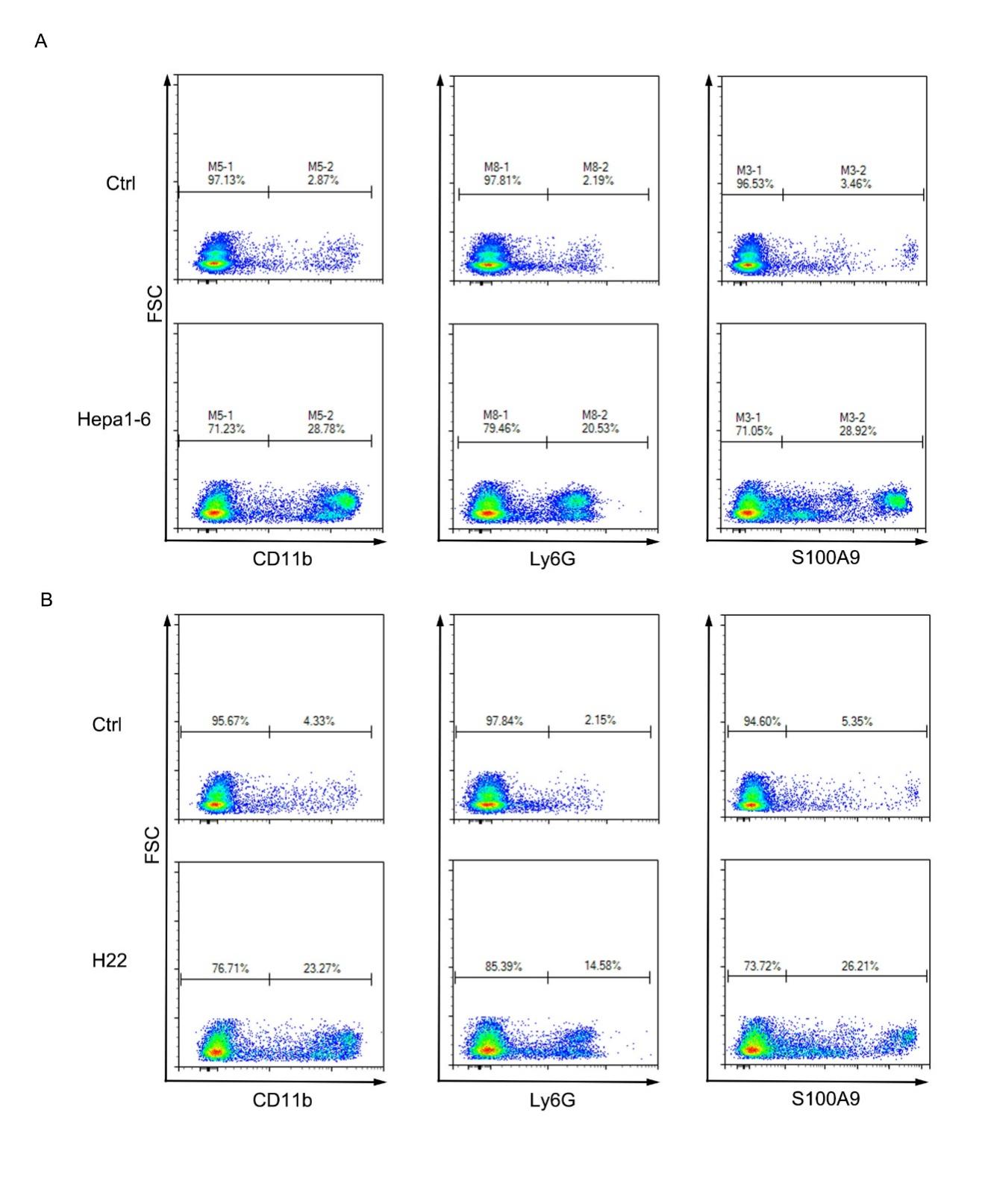


**Supplementary Figure 1. Increased numbers of myeloid cells and inflammatory cells in the pre-metastatic lung of orthotopic HCC xenografts.** Flow cytometry analysis of CD11b^+^, Ly6G^+^ and S100A9^+^ cells in the lung of mice 2 weeks after orthotopic implantation of Hepa1-6 cells or 1 week after orthotopic implantation of H22 cells in the liver.

**
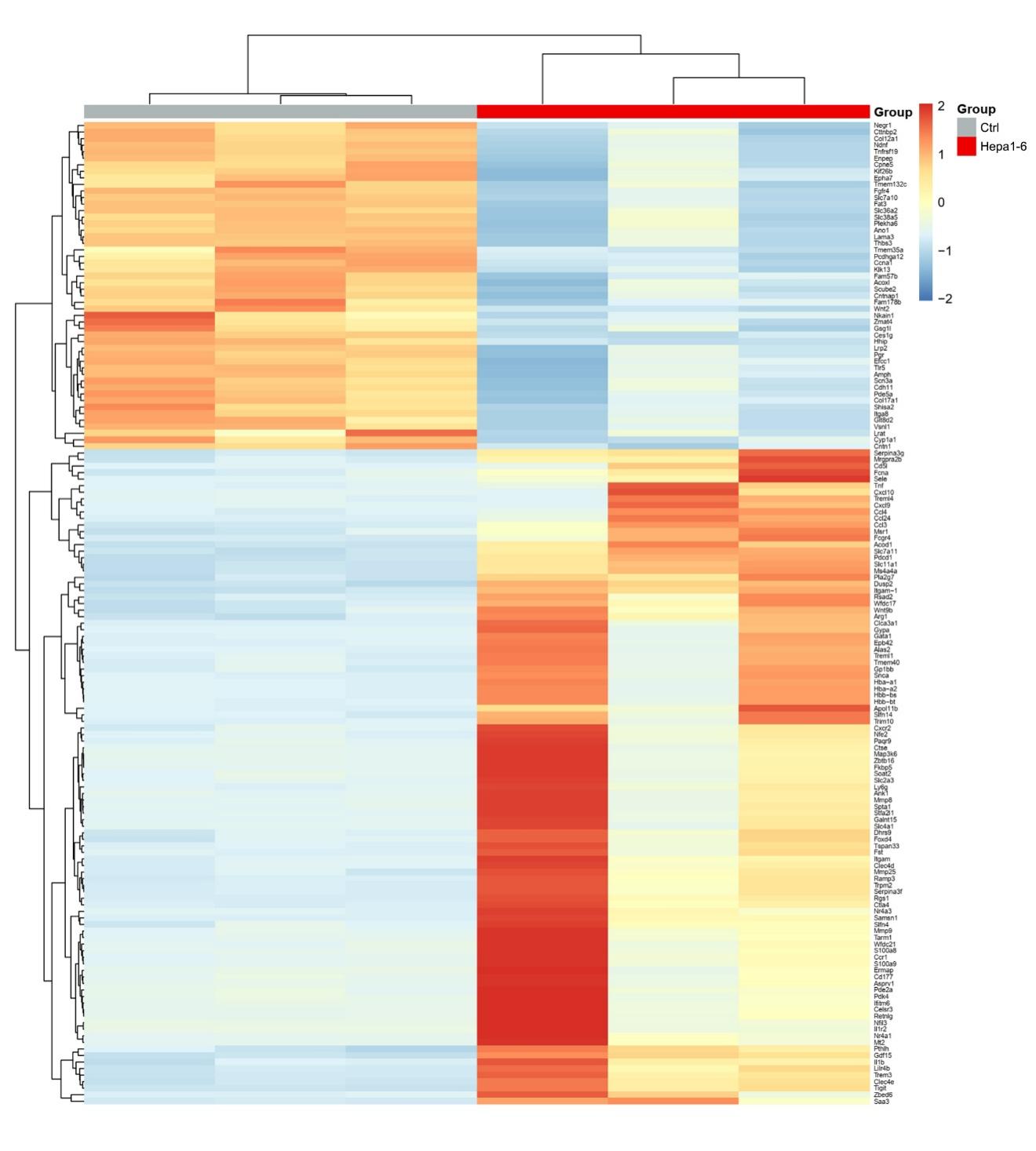
**

**Supplementary Figure 2. Heatmap showing dysregulated genes in the lung of mice 2 weeks after orthotopic implantation of Hepa1-6 cells or matrigel in the liver.**

**
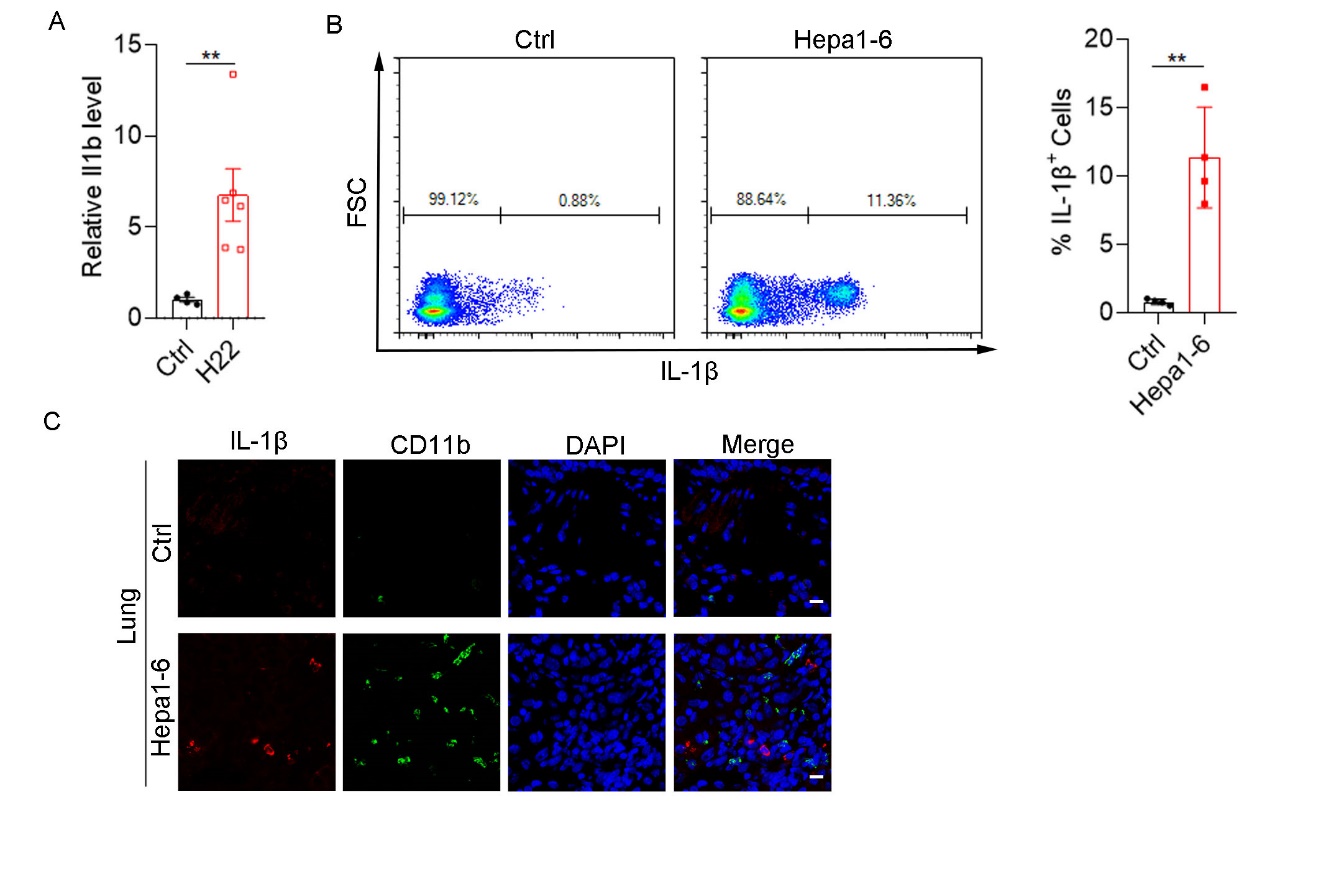
**

**Supplementary Figure 3. Il1b mRNA level was increased in the pre-metastatic lung of H22 xenograft.** (A) One week after orthotopic H22 implantation, Il1b mRNA level in the lung were detected by qPCR. (B) Flow cytometry analysis of IL-1β^+^ cells in the lung of mice bearing Hepa1-6 cells for 2 weeks. (C) IL-1β was not co-stained with CD11b in the pre-metastatic lung of Hepa1-6 xenograft. Double immunofluorescence staining of IL-1β (red) and CD11b (green) in the pre-metastatic lung. Scale bar, 10 μm. **, *P* < 0.01.

**
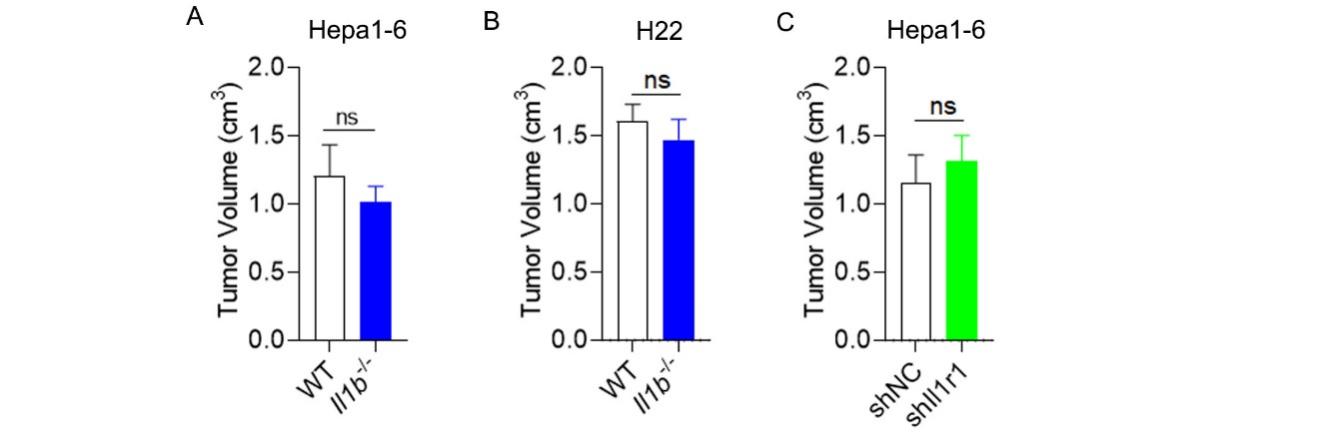
**

**Supplementary Figure 4. Tumor volume of orthotopic HCC xenograft in WT, *Il1b^-/-^* mice and mice infected with lenti-shNC or lenti-shIl1r1 viruses in the lung.** Ns, not significant.

**
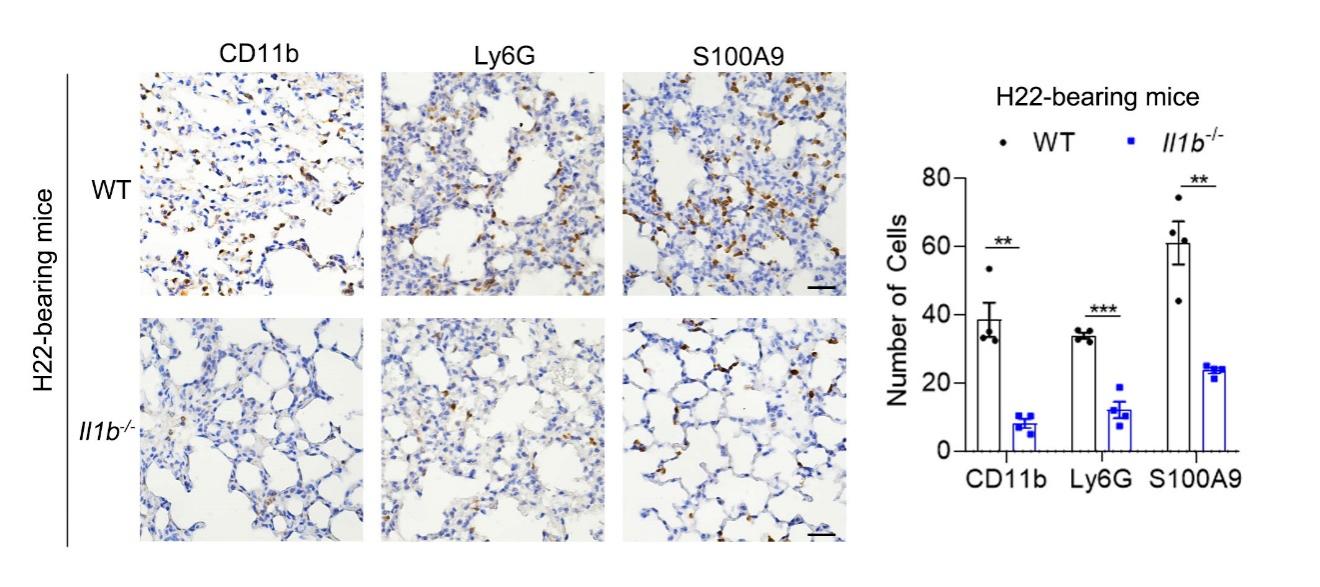
**

**Supplementary Figure 5. The numbers of CD11b^+^, Ly6G^+^ and S100A9^+^ cells in the pre-metastatic lung of H22 xenograft in** ***Il1b^-/-^* mice were significantly decreased compared with those in WT mice.** One week after H22 implantation, the lungs were harvested and applied to IHC staining. Scale bar, 25 μm. **, *P* < 0.01; ***, *P* < 0.001.

**
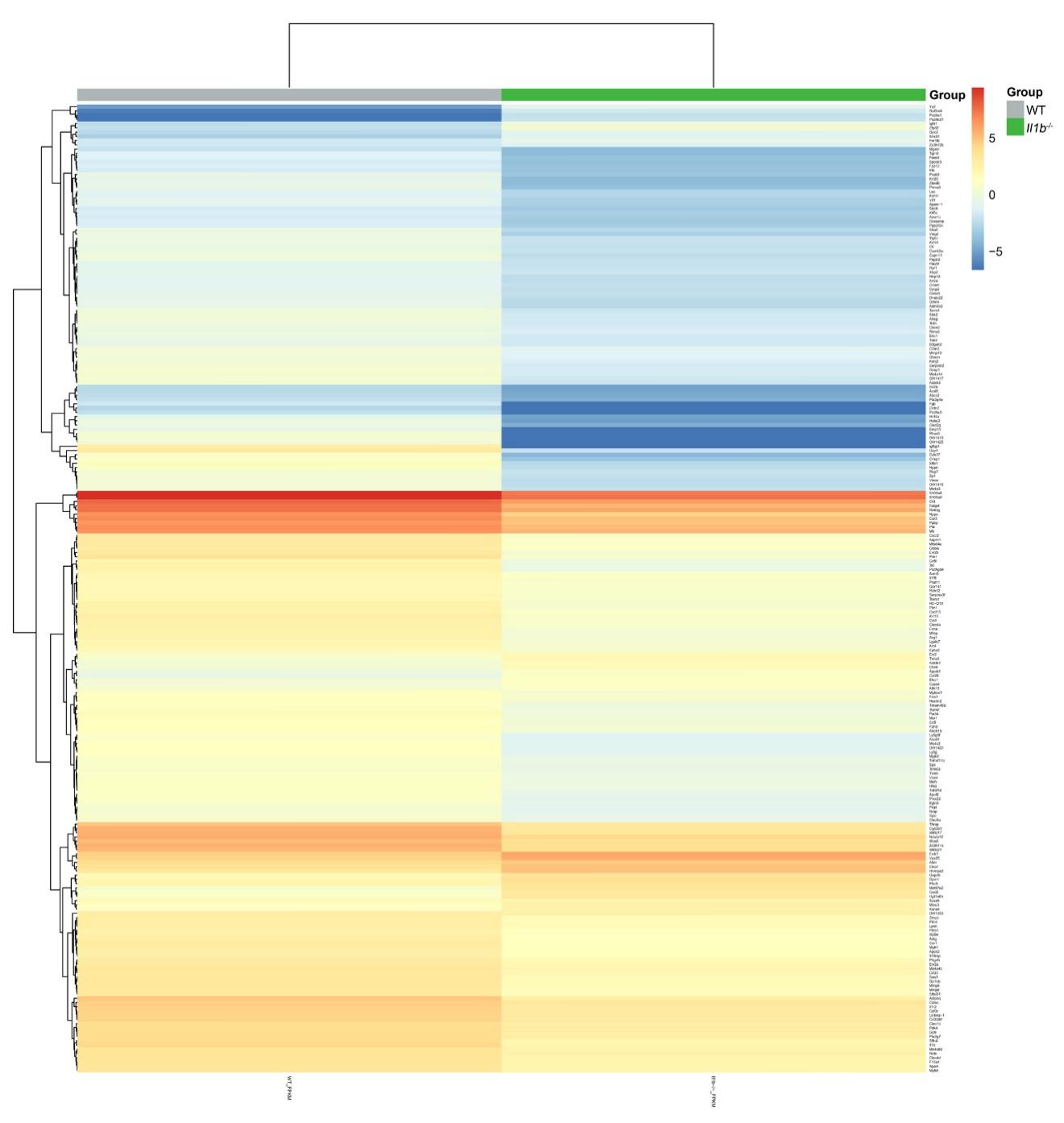
**

**Supplementary Figure 6. Heatmap showing dysregulated genes in the lung of mice 2 weeks after orthotopic implantation of Hepa1-6 cells in the liver of WT and *Il1b^-/-^* mice.**

**
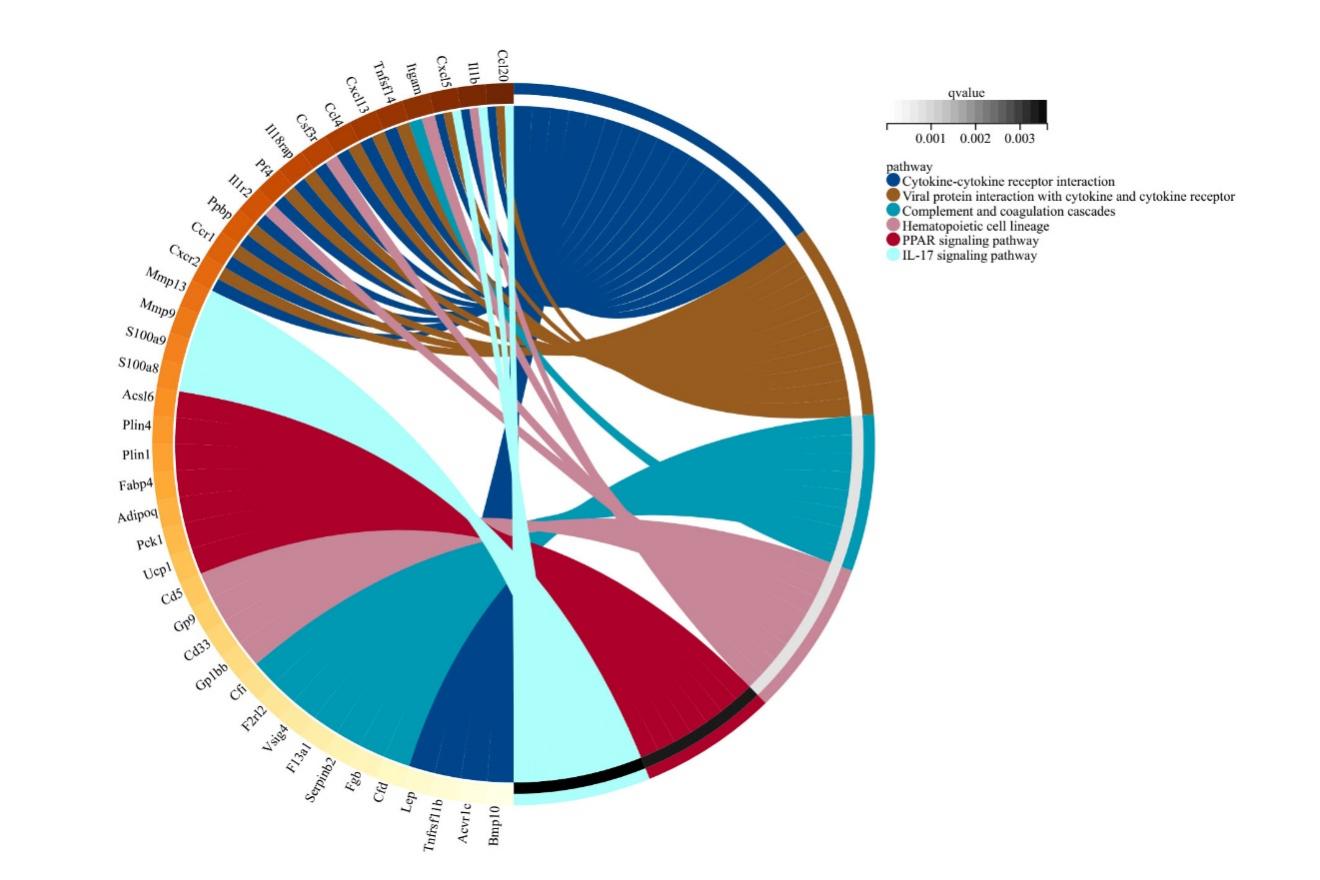
**

**Supplementary Figure 7. KEGG analysis of downregulated genes in the pre-metastatic lung of Hepa1-6 orthotopic xenograft in *Il1b^-/-^* mice compared with that in WT mice.**

**
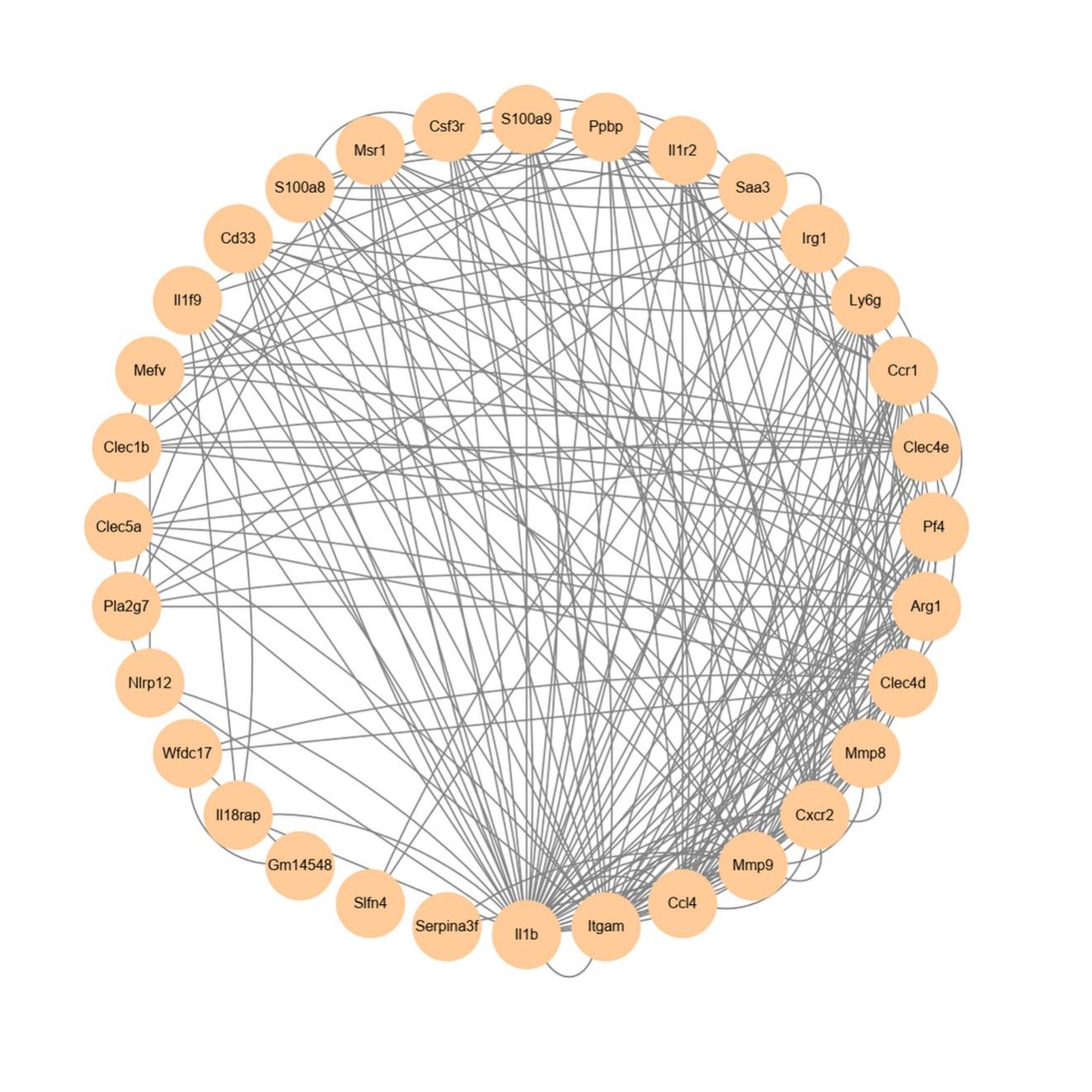
**

**Supplementary Figure 8. PPI analysis of IL-1β-regulated genes.** The network of the top one important module was visualized by cytoscape.

**
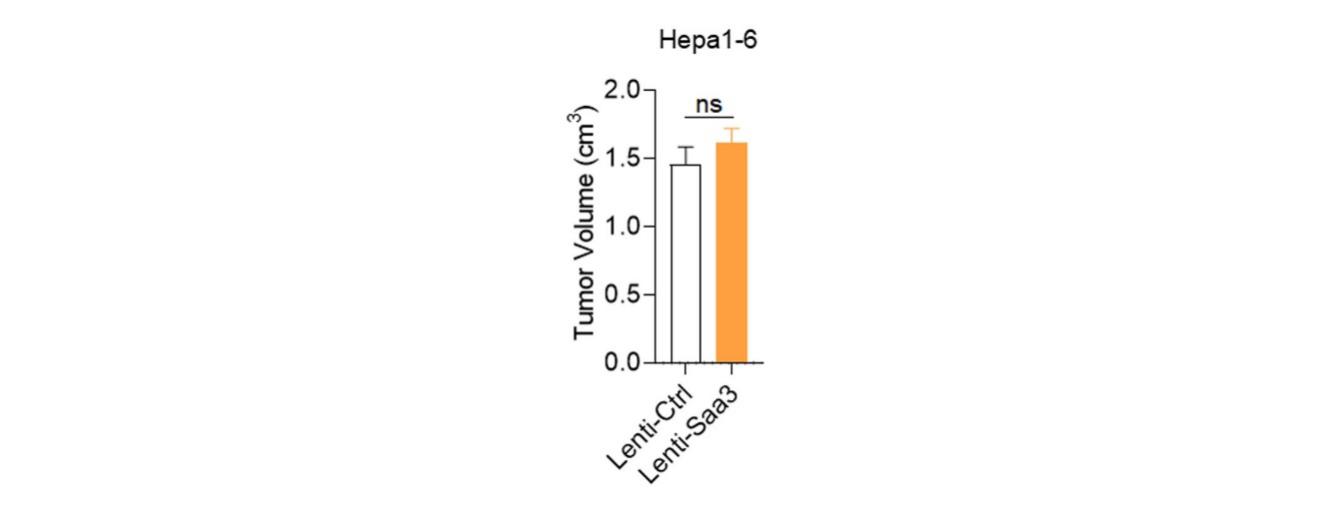
**

**Supplementary Figure 9. Overexpression of Saa3 in the lung didn’t affect primary tumor growth in *Il1b*^-/-^ mice.** Ns, not significant.


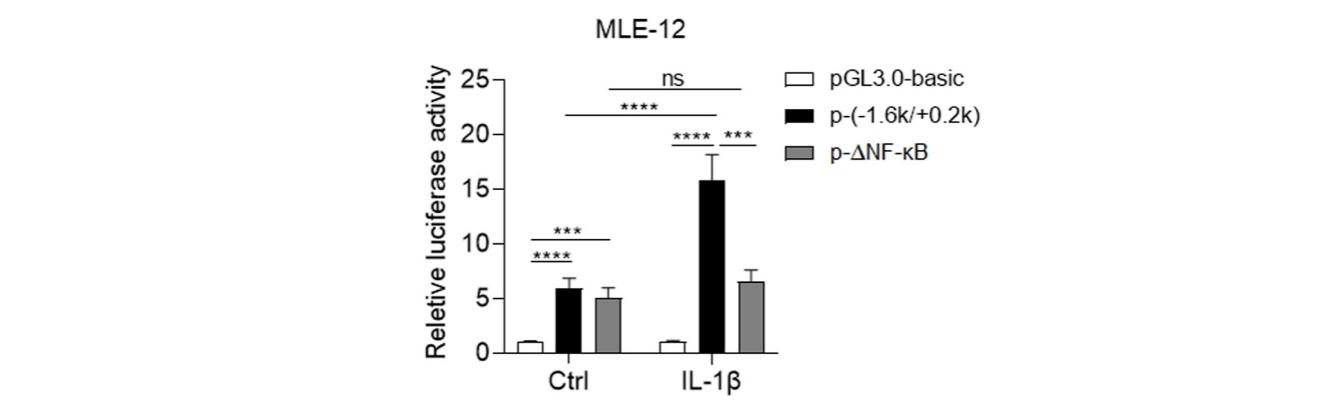


**Supplementary Figure 10. IL-1β increased the activity of Saa3 promoter through NF-κB.** Plasmid pRL-TK and pGL3.0-basic/p-(−1.6k/+0.2k)/p-∆NF-κB were co-transfected into MLE-12 cells in a 48-well plate. After 24 hours, cells were treated with or without IL-1β (10 ng/ml). Cells were harvested 24 hours later and luciferase activity was measured.

**
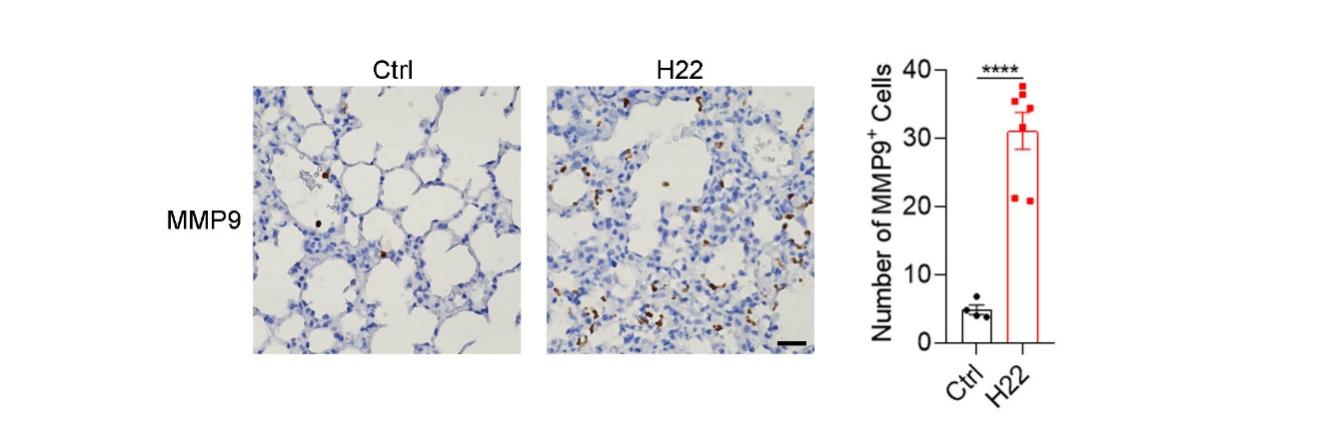
**

**Supplementary Figure 11. The number of MMP9^+^ cells was increased in the pre-metastatic lung of H22-bearing mice.** One week after orthotopic implantation of matrigel or H22 cells in the liver, lungs were harvested and subjected to IHC staining for MMP9. ****, *P* < 0.0001

**
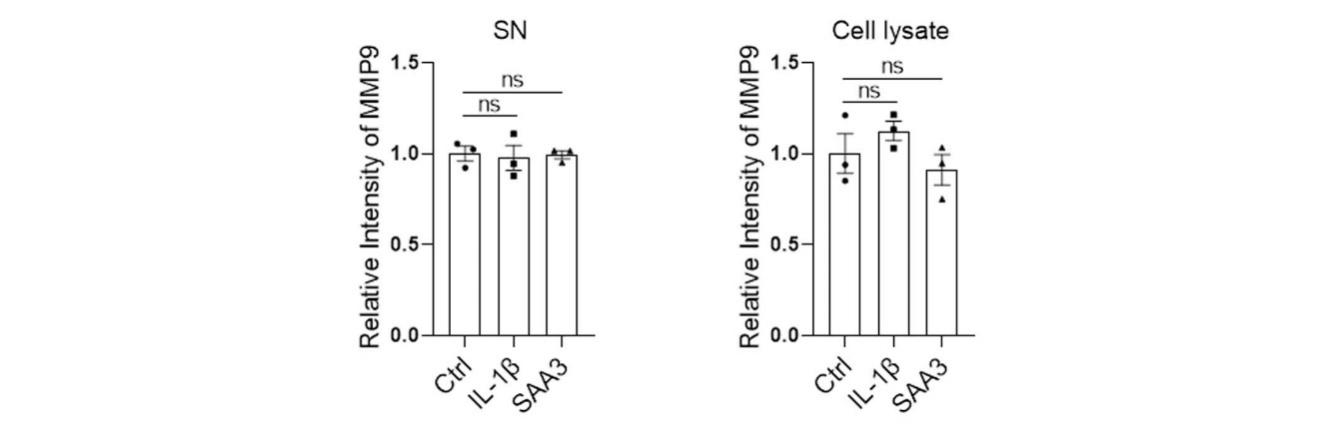
**

**Supplementary Figure 12. IL-1β and SAA3 stimulation had no effect on the protein level and secretion of MMP9 in BMDCs**. Mouse BMDCs were stimulated with IL-1β and SAA3 for 24 hours followed by immunoblotting analysis of MMP9 in cell lysate and supernatant (SN) of BMDCs. Relative intensity of each protein was quantified by Image J and shown above. Ns, not significant.

**
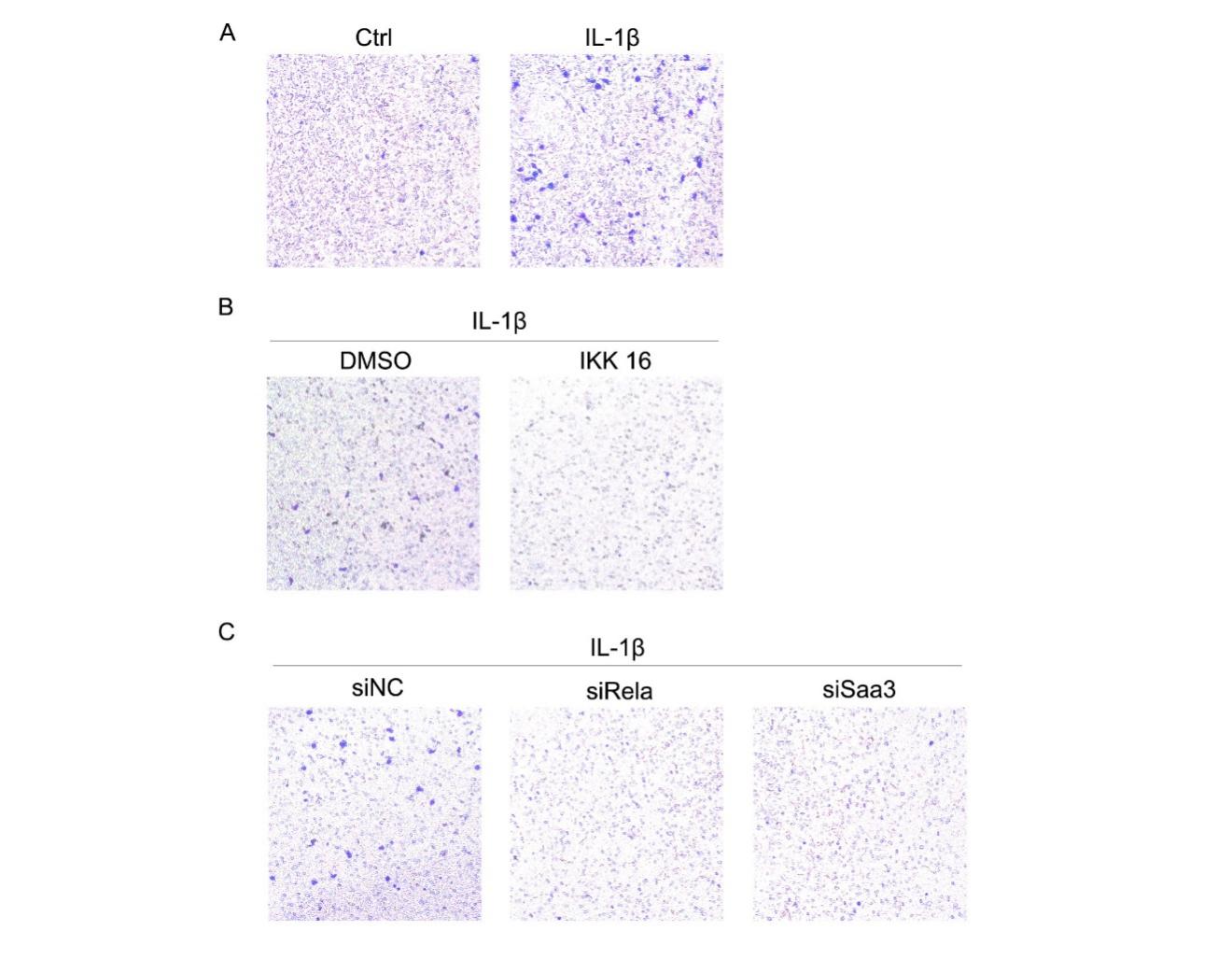
**

**Supplementary Figure 13. IL-1β enhanced the chemotactic activity of MCM via SAA3 in an NF-****κB dependent manner.** Representative images of migrated cells in Figure 6H-6J were shown above.

| **Supplementary Table 1. Sequences of shRNAs** | |
| --- | --- |
| **Name** | **Sequences** |
| shNC | CCTAAGGTTAAGTCGCCCTCGACCGGTCGAGGGCGACTTAACCTTAGG |
| shIl1r1 | CCGTACTTTCTGATGGTGTTTCTCGAGAAACACCATCAGAAAGTACGG |

| **Supplementary Table 2. Sequences of RNA and DNA Oligonucleotides** | | |
| --- | --- | --- |
| **Name** | **Sense strand/Sense primer (5'-3')** | **Antisense strand/Antisense primer (5'-3')** |
| **Primers for RT-PCR** | | |
| Il1b | CCTGTGTTTTCCTCCTTGCCT | GCCTAATGTCCCCTTGAATCAA |
| Luc2 | GAGGCTAAGGTGGTGGACTT | CCACGATGAAGAAGTGCTCG |
| Il1r1 | GTGCTACTGGGGCTCATTTGT | GGAGTAAGAGGACACTTGCGAAT |
| Saa3 | AACTATGATGCTGCCCGGAG | CAGCTCTTGAGTCCTCTGCT |
| Mmp9 | GCGTCGTGATCCCCACTTAC | CAGGCCGAATAGGAGCGTC |
| Rela | AGGCTTCTGGGCCTTATGTG | TGCTTCTCTCGCCAGGAATAC |
| Gapdh | AACTTTGGCATTGTGGAAGG | CACATTGGGGGTAGGAACAC |
|  |  |  |
| **siRNA duplexes** | | |
| siIl1r1 | GGUCCUUCUCAGACUUAGAdTdT | UCUAAGUCUGAGAAGGACCdTdT |
| siRela | CUCAAGAUCUGCCGAGUAAdTdT | UUACUCGGCAGAUCUUGAGdTdT |
| siSaa3 | CUGCUAAAGUCAUCAGCGAdTdT | UCGCUGAUGACUUUAGCAGdTdT |
| siNC | UUCUCCGAACGUGUCACGUdTdT | ACGUGACACGUUCGGAGAAdTdT |

**Supplementary Table 3. Differentially expressed genes in the premetastatic niche of orthotopic Hepa1-6 xenograft.** CL, Ctrl lung; TL, Hepa1-6 bearing lung.

**Supplementary Table4. Differentially expressed genes in the pre-metastatic lung of *Il1b^-/-^* mice compared with that of WT mice.**
